# Supplementary material for: Novel approach to estimate tuberculosis transmission in primary care clinics in sub-Saharan Africa: protocol of a prospective study
Source: BMJ Open. 2020 Aug 26;10(8):e036214. doi: 10.1136/bmjopen-2019-036214 (PMC7451471; doi:10.1136/bmjopen-2019-036214)
Supplement: Author's manuscript [file bmjopen-2019-036214.draft_revisions.pdf]

BMJ Open

BMJ Open is committed to open peer review. As part of this commitment we make the peer review history of every article we publish publicly available.

When an article is published we post the peer reviewers' comments and the authors' responses online. We also post the versions of the paper that were used during peer review. These are the versions that the peer review comments apply to.

The versions of the paper that follow are the versions that were submitted during the peer review process. They are not the versions of record or the final published versions. They should not be cited or distributed as the published version of this manuscript.

BMJ Open is an open access journal and the full, final, typeset and author-corrected version of record of the manuscript is available on our site with no access controls, subscription charges or pay-per-view fees (http://bmjopen.bmj.com).

If you have any questions on BMJ Open's open peer review process please email info.bmjopen@bmj.com

BMJ Open

A novel prospective study design to estimate tuberculosis transmission in primary care clinics in African high-burden settings

Journal:	*BMJ Open*
Manuscript ID	bmjopen-2019-036214
Article Type:	Protocol
Date Submitted by the Author:	05-Dec-2019

Complete List of Authors:	Zürcher, Kathrin; Universitat Bern Institut fur Sozial- und Praventivmedizin,<br>Morrow, Carl<br>Riou, Julien<br>Ballif, Marie; University of Bern, Bern, Switzerland, Institute of Social and Preventive Medicine<br>Koch, Anastazia<br>Bertschinger, Simon<br>Liu, Xin<br>Sharma, Manuja<br>Middelkoop, Keren ; Desmond Tutu/UCT, Cape Town, South Africa<br>Warner, Digby<br>Wood, Robin; University of Cape Town, Institute of Infectious Disease and Molecular Medicine and the Department of Medicine<br>Egger, Matthias; University of Bern, Institute of Social & Preventive Medicine<br>Fenner, Lukas; Universitat Bern,

Keywords:	Tuberculosis < INFECTIOUS DISEASES, INFECTIOUS DISEASES, PUBLIC HEALTH, PRIMARY CARE

SCHOLARONE™
Manuscripts

I, the Submitting Author has the right to grant and does grant on behalf of all authors of the Work (as defined in the below author licence), an exclusive licence and/or a non-exclusive licence for contributions from authors who are: i) UK Crown employees; ii) where BMJ has agreed a CC-BY licence shall apply, and/or iii) in accordance with the terms applicable for US Federal Government officers or employees acting as part of their official duties; on a worldwide, perpetual, irrevocable, royalty-free basis to BMJ Publishing Group Ltd ("BMJ") its licensees and where the relevant Journal is co-owned by BMJ to the co-owners of the Journal, to publish the Work in this journal and any other BMJ products and to exploit all rights, as set out in our licence.

The Submitting Author accepts and understands that any supply made under these terms is made by BMJ to the Submitting Author unless you are acting as an employee on behalf of your employer or a postgraduate student of an affiliated institution which is paying any applicable article publishing charge ("APC") for Open Access articles. Where the Submitting Author wishes to make the Work available on an Open Access basis (and intends to pay the relevant APC), the terms of reuse of such Open Access shall be governed by a Creative Commons licence – details of these licences and which Creative Commons licence will apply to this Work are set out in our licence referred to above.

Other than as permitted in any relevant BMJ Author's Self Archiving Policies, I confirm this Work has not been accepted for publication elsewhere, is not being considered for publication elsewhere and does not duplicate material already published. I confirm all authors consent to publication of this Work and authorise the granting of this licence.

For peer review only

TITLE PAGE *Journal: BMJ Open – Section "Study protocol"*

A novel prospective study design to estimate tuberculosis transmission in primary care clinics in African high-burden settings

Kathrin Zürcher [1], Carl Morrow [2], Julien Riou [1], Marie Ballif [1], Anastazia Koch [3], Simon Bertschinger [4], Xin Liu [5], Manuja Sharma [5], Keren Middelkoop [2], Digby F. Warner [3], Robin Wood [2], Matthias Egger [1, 6], Lukas Fenner [1]

1 Institute of Social and Preventive Medicine, University of Bern, Bern, Switzerland
2 Desmond Tutu HIV Centre, Institute of Infectious Disease and Molecular Medicine, University of Cape Town, University of Cape Town, Cape Town, South Africa
3 Molecular Mycobacteriology Research Unit, Institute of Infectious Disease and Molecular Medicine, University of Cape Town, Cape Town, South Africa.
4 Medical Informatics, Bern University of Applied Sciences, Bern, Switzerland
5 Paul G. Allen School of Computer Science & Engineering, University of Washington, Seattle, United States
6 Centre for Infectious Disease Epidemiology & Research, School of Public Health & Family Medicine, University of Cape Town, Cape Town, South Africa

Correspondence:

§ Lukas Fenner
Institute of Social and Preventive Medicine, University of Bern (ISPM)
Mittelstrasse 43, 3012 Bern, Switzerland
Phone: +41 31 631 35 18
E-Mail: lukas.fenner@ispm.unibe.ch

Key words: *Mycobacterium tuberculosis*, Tuberculosis, Transmission, CO_2

Running head: Estimating tuberculosis transmission

Word count, inserts and additional files:

Abstract 297 words, main text 2,637 words

Inserts: 1 table, 3 figures, 32 references

For peer review only

ABSTRACT

Introduction: Tuberculosis (TB) transmission is difficult to measure and its drivers are not well understood. The effectiveness of infection control measures at healthcare clinics and the most appropriate intervention strategies to interrupt transmission are unclear. We propose a novel approach using clinical, environmental and position-tracking data to study the risk of TB transmission at primary care clinics in TB and HIV high burden settings in sub-Saharan Africa.

Methods and analysis: We describe a novel and rapid study design to assess risk factors for airborne TB transmission at primary care clinics in high burden settings. The study protocol combines a range of different measurements. We will collect anonymous data on the number of patients, waiting times and patient movements using video sensors. In addition, we will collect acoustic sound recordings to determine the frequency and intensity of coughing. Environmental data will include indoor carbon dioxide levels (CO_2 in parts per million) and relative humidity. We will also extract routinely collected clinical data from the clinic records. The number of *Mycobacterium tuberculosis* particles in the air will be ascertained from dried filter units using highly sensitive digital droplet PCR. We will calculate rebreathed air volume based on people density and CO_2 levels and develop a mathematical model to estimate the risk of TB transmission. The mathematical model will be used to estimate the effect of interventions such as separating patient flows or improving ventilation in reducing transmission. The feasibility of our approach was recently demonstrated in a pilot study in a primary care clinic in Cape Town, South Africa.

Ethics and dissemination: The study has been approved by the University of Cape Town (HREC/REF no. 228/2019), and the City of Cape Town (ID-8139). The results will be disseminated in international peer-reviewed journals and presented at national and international conferences.

For peer review only - http://bmjopen.bmj.com/site/about/guidelines.xhtml

Strengths and limitations of this study:

- Tuberculosis (TB) transmission is difficult to measure and its drivers are not well understood.
- This protocol describes a novel approach to studying TB transmission using longitudinal study design in primary care clinics in high TB and HIV burden settings.
- This rapid approach will combine a wide range of different measurements, including patient waiting times and movements, acoustic recording of coughing, measurement of carbon dioxide levels and of *Mycobacterium tuberculosis* particles in the air.
- This study will lead to a precise and comprehensive transmission model to measure the effects of various interventions.
- Study limitations pertain to the need of power and stable WiFi over 24 hours for data collection at the primary care clinic, which is often an issue in low- and middle-income countries.

For peer review only

INTRODUCTION

Tuberculosis (TB), caused by the bacterium *Mycobacterium tuberculosis (Mtb)*, remains a major global public-health problem, particularly in the context of HIV. Sub-Saharan Africa is one of the most heavily burdened regions globally, although control measures have been in place since the beginning of the 20th century.[1] Over a century of investment in TB control has reduced TB mortality, but effective strategies are urgently needed to reduce TB transmission.[2] Drivers of the TB epidemic in sub-Saharan Africa are HIV-infection and the resulting immunodeficiency (the strongest risk factor),[1] delayed diagnosis and treatment as well as undetected and untreated cases of TB. These factors allow infectious TB patients to transmit *Mtb* to the community.[3-5] There are still many gaps in our knowledge on TB transmission such as the factors and locations associated with the risk of transmission, the effectiveness of infection control measures at clinics in high-burden settings, and the most appropriate intervention strategies to interrupt transmission.[5-7]

For TB transmission to occur, infected individuals must expel *Mtb* bacilli from their respiratory tract and an uninfected individual must inhale aerosols containing live bacilli to become infected. Transmission of *Mtb* is difficult to measure due to the lack of an *in vitro* test assay. The preferred approach is to measure presumptive transmission resulting in secondary cases as determined by molecular/genomic epidemiology. TB transmission has traditionally been investigated using contact tracing, analyses of geo-temporal clustering, and molecular typing.[8 9] However, molecular methods require resource-intensive culturing of strains and measure only transmission resulting in secondary cases. Furthermore, contact tracing is difficult to implement in resource-limited setting and may not be an effective control strategy in endemic areas where casual contacts are increasingly recognized as an important contributor to transmission.[10] New approaches are therefore urgently needed.

Mtb is carried in airborne particles (called infectious droplets) and these are generated when people with TB cough, sneeze or shout.[5] Indoor carbon dioxide (CO_2) levels have been used to assess the amount of exhaled air in a room, and the amount of rebreathed air.[11-13] Viable *Mtb* particles have been captured from contaminated air.[11 14-16] We describe a unique study design to assess risk factors for airborne TB transmission in primary care clinics in high TB/HIV burden settings which combines a range of relevant measurements, including data on patient and

For peer review only - http://bmjopen.bmj.com/site/about/guidelines.xhtml

infrastructure, movements of patients through the facility, coughing, environmental indoor CO_2 levels, and concentration of *Mtb* particles in the air.

For peer review only

For peer review only - http://bmjopen.bmj.com/site/about/guidelines.xhtml

METHODS

Study design

Longitudinal study with data collection at the levels of the patients, the clinic, and the environment.

Study setup

Primary care clinics in regions with a high TB and HIV burden in sub-Saharan Africa. A description and floor plan of an exemplary primary clinic indicating the planned study activities is shown in Figure 1.

Patient-level variables

Video sensor data

A person-tracking sensor system developed by Xovis (Zollikofen, Switzerland and Cambridge, MA; see www.xovis.com) will be used to monitor the clinic attenders' movements, which will be transformed into waiting times until medical consultation, number of people in different locations, highly frequented areas in the clinic, and average distance between people. To cover the target area, several sensors need to be installed, calibrated and validated. Sensors with overlapping ranges will be combined for the seamless coverage of people's movements over large areas (example shown in Figure 2). The raw data consist of the position (x-y coordinates captured every 0.25 seconds) for each unique individual during the entire duration of their stay within the clinic.

The sensors have four levels of privacy. In our study, patient confidentiality will be set on Privacy Level 2, a level that does not store images of the persons; only moving dots can be seen (see "Data Privacy and Security" by Xovis).[17 18]

Patient data

We will extract routinely collected patient clinical data from the patient including date and time of patient registration, age, sex, HIV status, TB diagnostic test results, mode of TB diagnosis (sputum smear microscopy, Xpert MTB/RIF, mycobacterium culture), date of TB treatment start, current anti-TB treatment regimen). In addition, we will collect site-level data such as setting, level of care, size of the clinic, adult/pediatric care provision, and infection control measures in place.

For peer review only - http://bmjopen.bmj.com/site/about/guidelines.xhtml

Definitions

Presumptive TB refers to a patient who presents with clinical symptoms or signs suggestive of TB. A bacteriologically confirmed TB case is a person for whom a biological specimen is positive by smear, microscopy, culture or rapid diagnostic such as Xpert MTB/RIF or line probe assay (LPA).[19]

Environmental variables

CO_2, relative humidity and temperature monitoring

CO_2 monitors (XEAST Digital CO2 Monitor XE-2000 Multifunction Gas Analyzer) will be used as previously described.[14 15] The monitors record indoor CO_2 concentrations (in parts per million [ppm]), temperature, and relative humidity (RH) minute by minute. Humidity is associated with *Mtb* survival in the air.[20] The monitors auto-calibrate over time to a standard minimum value of 400 ppm. This value is close to the average monthly outdoor carbon dioxide concentration measured at Cape Point, South Africa (405.48 ppm in December 2018).[21] The monitors will run for one to two weeks prior to sampling to allow them to calibrate and for quality assurance.

Cough monitoring

We will install a microphone (RØDE NT-USB, Sydney, Australia) near the ceiling to continuously record the sounds in the waiting room. *CoughSense*, a deep learning cough detection algorithm based on MXNet (open-source deep learning software framework) was developed to classify audio signals as coughing or other sounds.[22] The algorithm uses spectrograms extracted from the raw audio for classification. The model was trained and tested using multiple audio recordings obtained through clinical and ambulatory deployments. After identification of coughs in audio samples, we will extract information such as frequency, intensity and duration of coughing. The frequency of coughing will be calculated as the number of coughs per hour. The power spectrum of the audio sample will be analyzed to estimate the intensity profile of the cough signal. Length of the cough audio recording will be used to estimate the duration of each cough. To guarantee patient confidentiality the acoustic recordings of voices and other sounds will be deleted, while preserving the cough signals.

For peer review only - http://bmjopen.bmj.com/site/about/guidelines.xhtml

Bio-aerosol sampling and molecular detection

We will collect *Mtb* particles from the air using mobile bio-aerosol sampling devices (Dry Filter Unit [DFU]-1000, Lockheed Martin Integrated Systems, Gaithersburg, MD, USA). One DFU will be placed in the waiting room and the other in the TB treatment room. Each DFU collects air for 7 hours (two periods of 3.5 hours) every day onto two 1 µm polyester-felt filters. Duplicate filters from each sampling session will be processed by vortexing in sterile PBS (with 0.05% Tween 80) followed by centrifugation at 3750 rpm for 15 mins as previously described.[15] DNA from *Mtb* cells will be extracted using an in-house lysis buffer with subsequent pelleting (centrifugation 13000 rpm for 10 mins) of DNA and resuspension in 50 µl of Tris-EDTA buffer (10mM Trist, 1mM EDTA, pH 8.0). Given that ddPCR is relatively robust against inhibitors, no further DNA purification will be required. The primer/probe combinations and reaction conditions for *Mtb* specific ddPCR have been described.[15] In this study, samples with known amounts of purified *Mtb* DNA (0.01ng and 0.001ng) will be included to serve as positive internal controls for the ddPCR and nuclease free water will be included as negative control. The data generated from the ddPCR reaction will be analyzed via the Umbrella pipeline, [23] using only wells for which a minimum of 10,000 droplets had been detected.

Statistical analyses

We will describe and analyze the data separately by type and source of data and then use all data to parameterize a mathematical model.

1. *Patient data*: Numbers and characteristics of patients consulting the clinic overall will be summarized using descriptive statistics.
2. *Video sensor data:* Raw data about patients' movements will be transformed into waiting times until medical consultation, number of patients in the different locations, highly frequented areas in the clinic and average distance between patients (i.e. clustering). We will link each tracked individual to the clinical data collected from the clinic's database (i.e. TB/HIV diagnosis), using the order of arrival and time of registration.
3. *CO_2 data*: Measurements of CO_2 concentration at the different locations in the clinic (waiting room, registration desk, TB treatment room), together with estimates in outdoor air, will be used to estimate the proportion of air in the different locations that was expired by individuals, the *rebreathed fraction*.[12] We

For peer review only - http://bmjopen.bmj.com/site/about/guidelines.xhtml

will use a modified Wells-Riley model appropriate for non-steady states conditions of ventilation and number of individuals to describe and calculate the shared rebreathed air in the different locations in either liter/minute or liter/day and the air exchange (liter/hour per person). Table 1 describes the parameters to calculate the shared rebreathed air and the air exchange.[13 15 24]

The proportion of rebreathed air (*f*) will be calculated from the excess CO_2 measured indoors, divided by the exhaled CO_2 *(Ca)*:

Equation 1:

$$f = \frac{(C - Co)}{Ca}$$

(see Table 1 for definitions of parameters in this and the following equations).

Before we can calculate the rebreathed air volume (RAV) we will need the rebreathed proportion from other people (fo). Therefore we need to know the number of people *(n)* present at the each time point at the clinic:

Equation 2:

$$fo = f \times \frac{(n-1)}{n}$$

Finally we can calculate the RAV for each minute by multiplying fo and the minute respiratory volume (8 liters per minute, *[p]*) as:

Equation 3

$$Rebreathed\ air\ volume\ (RAV) = (pfo)$$

In a next step we aim to calculate the ventilation rate (air exchange). The indoor CO_2 generated rate is the product of the average volume of gas exhaled per person (0.13 l/s per person, *[V]*) and the CO_2 concentration of the exhaled air:

Equation 4:

$$Indoor\ CO_2\ generation\ rate\ in\ l/s\ per\ person\ (G) = VxCa$$

The ventilation rate is expressed as:

Equation 5:

$$Ventilation\ rate\ in\ l/s\ per\ person\ (Q) = \frac{G}{C - Co}$$

For peer review only - http://bmjopen.bmj.com/site/about/guidelines.xhtml

To calculate the air exchange per hour (ACH, *Equation 6*) we need to know the volume of air in a given location.

4. *Relative humidity (RH) and temperature:* We will describe changes of the RH and temperature over the day.
5. *Cough sounds*: Frequency, intensity, duration of recorded coughs per time period.[25 26]
6. *Detection of Mtb in bio-aerosol sampling*: The number of *Mtb* genome copies present in each sample.

Mathematical modelling

Following the descriptive analyses, we will develop a mathematical model (Figure 3) integrating all sources of data to describe the risk of TB transmission and identify key drivers of transmission.[13 24 27 28] We hypothesize that the number of individuals present, the rebreathed fraction and the frequency of coughs will have the greatest influence on the risk. As we do not actually observe transmission events, we will use *yu*, the number of *Mtb* genomes counted by the bio-aerosol sampling for each 6-hour period *u*, as the dependent variable. The model will describe *yu* as a Poisson process with time-dependent intensity *λt* (also called a Cox process). This intensity can be interpreted as a proxy for the (unobserved) risk of TB transmission. To harmonize over the different time structures of the collected data, *λtu* will be modelled using smaller, 10 minutes time periods *t* within *u*. In a first simple model, *λtu* will integrate multiple independent variables *xtu*: the number of individuals in the waiting room during time *t*, the rebreathed fraction and the number of coughs. The model will be estimated with MCMC in a Bayesian framework using *Stan*, a probabilistic programming language.[29]

Several metrics will be used to assess the goodness of fit of the model. We will then add more complexity to the model by integrating other independent variables such as the characteristics of the patients, shared rebreathed air (CO_2 monitors),[12-15] patient flow (clustering of individuals, movements of patients), and other potential drivers (intensity/frequency/duration of coughs).[25 26]

Having obtained a model of the risk of TB transmission, we will evaluate the potential effect of interventions. We aim to lower this risk by removing one or several drivers such as limiting the number of patients in the waiting room to a maximum;

For peer review only - http://bmjopen.bmj.com/site/about/guidelines.xhtml

patients with certain characteristics (e.g., history of TB) or coughers will be separated for the other patients or will be given face masks, and the waiting room is ventilated regularly. All analyses will be performed in R (version 3.6.0) and/or STATA (version 15.1, Stata Corporation, Texas, USA).

Feasibility of the approach: pilot study

We conducted a pilot study in Cape Town (South Africa) to demonstrate the feasibility of our approach (Figure 1). The pilot study took place at a primary care clinic, which incorporates HIV counseling and testing, and a TB clinic for the diagnosis and management of TB. The clinic is situated in a large settlement of formal and semi-formal housing in Cape Town, which has previously been described.[30 31] The clinic is open on workdays from Monday till Friday from 7 am till 4 pm (opening hours of the clinic). We studied clinic activities over four weeks on workdays between July 25 and August 23 2019. A stable power and WiFi over 24 hours during the whole study period is necessary for the data collection. To avoid power cuts in the future, power banks for devices such as laptops and WiFi routers could bridge these time gaps. However, the use of UPS power stabilizer and access to generator as back-up power may be needed for other devices such as CO_2 monitors.

Expansion of the study may include clinics in Cape Town, including the pilot study site, but also at other clinics in the Southern African region (Zambia, Zimbabwe, Malawi), including urban and rural sites to ensure representative and generalizable results.

Patient and Public Involvement

No patient directly involved.

Ethics statement

This study has been approved by the University of Cape Town Faculty of Health Sciences Human Research Ethics Committee (HREC/REF: 228/2019) and the City of Cape Town (Project ID 8139).

For peer review only - http://bmjopen.bmj.com/site/about/guidelines.xhtml

DISCUSSION

TB control is particularly relevant for sub-Saharan Africa, which carries a disproportionally large portion of the global burden of both TB and HIV. There is an urgent need to understand the drivers of TB transmission to begin reversing TB incidence using new intervention approaches.[1] A better understanding of transmission coupled with a rapid test system are likely to contribute to improving TB control in clinic settings. This project is likely to provide new insights into the complex TB transmission framework at a primary care clinic in an endemic setting using clinical data, CO_2 levels, cough analyses, video tracking and *Mtb* particles sampled form the air. Although, the implementation and evaluation of interventions are beyond the scope of this study, the results will generate new hypotheses and opportunities for public health intervention studies (e.g., randomized controlled or cluster-randomized trials). Importantly, with mathematical models based on real-life data, we can evaluate the likely effect of interventions thus improving intervention studies, and inform help with logistical and infrastructural planning of primary care clinics to reduce the transmission risk. Having established feasibility in one clinic in South Africa, we are planning to collect data in several countries in sub-Saharan Africa. The results from this broader study have the potential to inform national and international guidelines to reduce TB transmission at health care centers.

Strengths and limitations: This is a novel and rapid approach to studying TB transmission combining a wide range of different measurements, which goes beyond the traditional approaches such as contact tracing, geotemporal clustering or molecular genotyping. It will lead to a precise and comprehensive transmission model to measure the effects of various interventions, paving the way for future studies. Study limitations pertain to the need of power and stable WiFi over 24 hours for data collection at the primary care clinic which is often an issue in low- and middle-income countries. However, this can be addressed by using UPS power stabilizer and with access to generator back-up power as tested during the pilot work.

DISSEMINATION

The results will be disseminated in international peer-reviewed journals and presented at national and international conferences.

AUTHORS CONTRIBUTIONS

For peer review only - http://bmjopen.bmj.com/site/about/guidelines.xhtml

Overall concept: KZ, CM, MB, RW, ME, LF. Medical informatics: SB and JR. Cough sound concept: CM XL RW. Video sensor concept: KZ JB SB ME LF. Patient data concept: CM KM RW. Laboratory work concept: CM AK KM DW RW. Modelling work concept: KZ JR SB ME LF. Pilot study coordination: CM KM RW.

ACKNOWLEDGEMENTS

We would like to thank Mbali Mohlamonyane for setting up the bio-aerosol sample collection system, Zeenat Hoosen and Ronnett Seldon for developing the laboratory protocols. We are also grateful to the clinical manager and the staff members at the pilot clinic. Finally, we would like to thank for the support from the City of Cape Town for the use of one of their clinic facilities. Research findings and recommendations do not represent an official view of the City of Cape Town.

SOURCES OF SUPPORT

The roll-out of this study is supported by a grant from the Swiss National Science Foundation (grant no. CRSK-3_190781). KZ and LF are supported by the National Institute of Allergy and Infectious Diseases (NIAID) through grant no. 5U01-AI069924-05, ME by special project funding from the Swiss National Science Foundation (grant no. 174281).

COMPETING INTERESTS

All authors declare that they have no conflicts of interest.

For peer review only - http://bmjopen.bmj.com/site/about/guidelines.xhtml

REFERENCES

1. World Health Organization. Global tuberculosis report 2018. *World Health Organization Document* 2018;WHO/HTM/TB/2018:277.

2. Hermans S, Horsburgh CR, Jr., Wood R. A Century of Tuberculosis Epidemiology in the Northern and Southern Hemisphere: The Differential Impact of Control Interventions. *PloS one* 2015;10(8):e0135179. doi: 10.1371/journal.pone.0135179

3. Wood R, Lawn SD, Caldwell J, et al. Burden of new and recurrent tuberculosis in a major South African city stratified by age and HIV-status. *PloS one* 2011;6(10):e25098. doi: 10.1371/journal.pone.0025098

4. The Lancet Respiratory M. Tuberculosis: a much-needed focus on transmission. *Lancet Respir Med* 2019;7(6):465. doi: 10.1016/S2213-2600(19)30148-1

5. Rieder HL. Epidemiologic basis of tuberculosis control. In: International Union Against Tuberculosis and Lung Disease, ed. 1 ed. Paris: International Union Against Tuberculosis and Lung Disease, 1999:1-162.

6. Lin HH, Dowdy D, Dye C, et al. The impact of new tuberculosis diagnostics on transmission: why context matters. *Bull World Health Organ* 2012;90:739-47A.

7. Middelkoop K, Mathema B, Myer L, et al. Transmission of tuberculosis in a South African community with a high prevalence of HIV infection. *J Infect Dis* 2015;211:53-61.

8. Walker TM, Ip CL, Harrell RH, et al. Whole-genome sequencing to delineate *Mycobacterium tuberculosis* outbreaks: a retrospective observational study. *Lancet Infect Dis* 2012 doi: S1473-3099(12)70277-3 [pii];10.1016/S1473-3099(12)70277-3

9. Cook VJ, Shah L, Gardy J, et al. Recommendations on modern contact investigation methods for enhancing tuberculosis control. *The international journal of tuberculosis and lung disease* 2012;16:297-305.

10. Middelkoop K, Bekker LG, Morrow C, et al. Decreasing household contribution to TB transmission with age: a retrospective geographic analysis of young people in a South African township. *BMC infectious diseases* 2014;14:221. doi: 10.1186/1471-2334-14-221

11. Andrews JR, Morrow C, Walensky RP, et al. Integrating social contact and environmental data in evaluating tuberculosis transmission in a South African township. *J Infect Dis* 2014 doi: 10.1093/infdis/jiu138

For peer review only - http://bmjopen.bmj.com/site/about/guidelines.xhtml

12. Myatt TA, Johnston SL, Rudnick S, et al. Airborne rhinovirus detection and effect of ultraviolet irradiation on detection by a semi-nested RT-PCR assay. *BMC Public Health* 2003;3:5.
13. Rudnick SN, Milton DK. Risk of indoor airborne infection transmission estimated from carbon dioxide concentration. *Indoor Air* 2003;13(3):237-45.
14. Wood R, Morrow C, Ginsberg S, et al. Quantification of shared air: a social and environmental determinant of airborne disease transmission. *PloS one* 2014;9(9):e106622. doi: 10.1371/journal.pone.0106622
15. Richardson ET, Morrow CD, Kalil DB, et al. Shared air: a renewed focus on ventilation for the prevention of tuberculosis transmission. *PloS one* 2014;9(5):e96334. doi: 10.1371/journal.pone.0096334
16. Patterson B, Morrow C, Singh V, et al. Detection of Mycobacterium tuberculosis bacilli in bio-aerosols from untreated TB patients. *Gates Open Res* 2017;1:11. doi: 10.12688/gatesopenres.12758.1
17. Xovis. Guaranteed data privacy Zollikofen2018 [Available from: https://www.xovis.com/technology/detail/guaranteed-data-privacy/ accessed 03.12.2019 2019.
18. Xovis. Xovis' Privacy Policy Zollikofen2018 [Available from: https://www.xovis.com/data-privacy/ accessed 03.12.2019 2019.
19. World Health Organization. Definitions and reporting framework for tuberculosis–2013 revision: World Health Organization 2013.
20. Sornboot J, Aekplakorn W, Ramasoota P, et al. Detection of airborne Mycobacterium tuberculosis complex in high-risk areas of health care facilities in Thailand. *The international journal of tuberculosis and lung disease* 2019;23(4):465-73. doi: 10.5588/ijtld.18.0218
21. National Oceanic & Atmospheric Administration. Earth System Research Laboratory; Global Monitoring Division [updated 09.11.2019. Available from: https://www.esrl.noaa.gov/gmd/dv/data/index.php?site=CPT&parameter_name=Carbon%2BDioxide,%20accessed%2012%20November%202019 accessed 12.11.2019 2019.
22. Chen T, Li M, Li Y, et al. Mxnet: A flexible and efficient machine learning library for heterogeneous distributed systems. *arXiv preprint arXiv:151201274* 2015

For peer review only - http://bmjopen.bmj.com/site/about/guidelines.xhtml

23. Jacobs BKM, Goetghebeur E, Vandesompele J, et al. Model-Based Classification for Digital PCR: Your Umbrella for Rain. *Analytical chemistry* 2017;89(8):4461-67. doi: 10.1021/acs.analchem.6b04208
24. Riley RL, Mills CC, Nyka W, et al. Aerial dissemination of pulmonary tuberculosis. A two-year study of contagion in a tuberculosis ward. 1959. *Am J Epidemiol* 1995;142(1):3-14. doi: 10.1093/oxfordjournals.aje.a117542
25. Birring SS, Fleming T, Matos S, et al. The Leicester Cough Monitor: preliminary validation of an automated cough detection system in chronic cough. *Eur Respir J* 2008;31(5):1013-8. doi: 10.1183/09031936.00057407
26. Botha GHR, Theron G, Warren RM, et al. Detection of tuberculosis by automatic cough sound analysis. *Physiol Meas* 2018;39(4):045005. doi: 10.1088/1361-6579/aab6d0
27. Gammaitoni L, Nucci MC. Using a mathematical model to evaluate the efficacy of TB control measures. *Emerg Infect Dis* 1997;3(3):335-42. doi: 10.3201/eid0303.970310
28. Issarow CM, Mulder N, Wood R. Modelling the risk of airborne infectious disease using exhaled air. *Journal of Theoretical Biology* 2015;372(0):100-06. doi: http://dx.doi.org/10.1016/j.jtbi.2015.02.010
29. Carpenter J, Crutchley P, Zilca RD, et al. Correction: Seeing the "Big" Picture: Big Data Methods for Exploring Relationships Between Usage, Language, and Outcome in Internet Intervention Data. *J Med Internet Res* 2017;19(12):e347. doi: 10.2196/jmir.8099
30. Wood R, Middelkoop K, Myer L, et al. Undiagnosed tuberculosis in a community with high HIV prevalence: implications for tuberculosis control. *Am J Respir Crit Care Med* 2007;175(1):87-93.
31. Middelkoop K, Bekker LG, Myer L, et al. Antiretroviral therapy and TB notification rates in a high HIV prevalence South African community. *Journal of acquired immune deficiency syndromes (1999)* 2011;56(3):263-9. doi: 10.1097/QAI.0b013e31820413b3
32. Emmerich SJ, Persily AK. State-of-the-art review of CO2 demand controlled ventilation technology and application: Citeseer 2001.

For peer review only

For peer review only - http://bmjopen.bmj.com/site/about/guidelines.xhtml

TABLES AND FIGURE LEGENDS

Table 1: Description of the parameter to calculate the shared rebreathed air volume and air exchange as well as parameters to construct the mathematical transmission model (see Figure 3) and equations 1 to 6 in the main text. Adapted from Richardson et al.[15]

Parameter	Description	Value	Reference
C	Observed CO_2 concentration in the indoor air per minute	observed	-
Co	CO_2 concentration in the outdoor air per minute	400-420ppm	21
Ca	CO_2 concentration in the exhaled air	38000-40000ppm	13, 32
f	Proportion of rebreathed air	equation	-
n	Number of people recorded at the location	observed	-
fo	Rebreathed proportion from other people	equation	-
p	Minute respiratory volume	8 l/per minutes	32
RAV	Rebreathed air volume	equation	-
V	Average volume of gas exhaled per person	0.13 l/s per person	15
G	Indoor CO_2 generation rate (l/s per person)	equation	-
Q	Ventilation rate (l/s per person)	equation	-
vol	Volume of the room	calculated	-

For peer review only - http://bmjopen.bmj.com/site/about/guidelines.xhtml

FIGURE LEGENDS

Figure 1: Floor plan of the pilot primary care clinic in Cape Town, South Africa, indicating study measurements.

Figure 2: Tracked persons as "moving dots" using Xovis video sensor. Three different video sectors are merged together (pilot study).

Figure 3: Model structure.

For peer review only

For peer review only - http://bmjopen.bmj.com/site/about/guidelines.xhtml

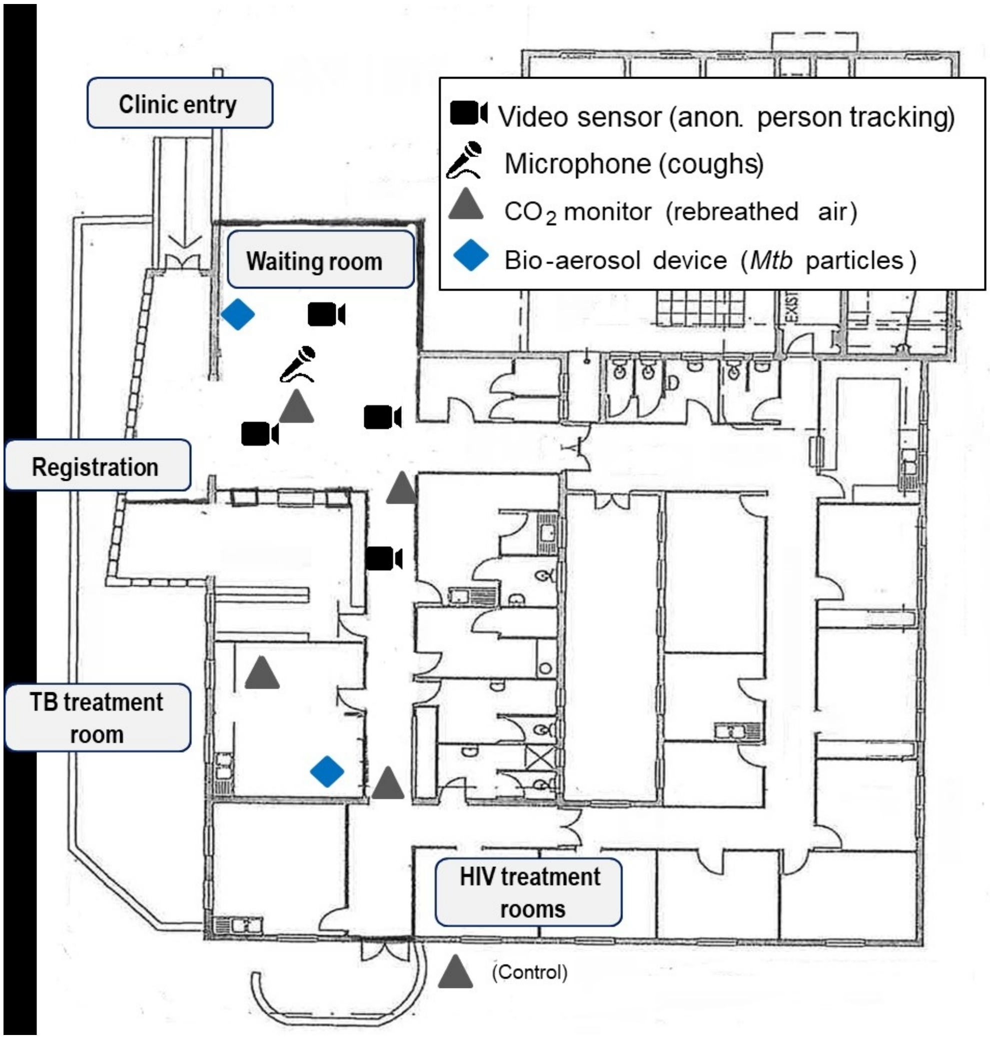

Figure 1

163x167mm (150 x 150 DPI)

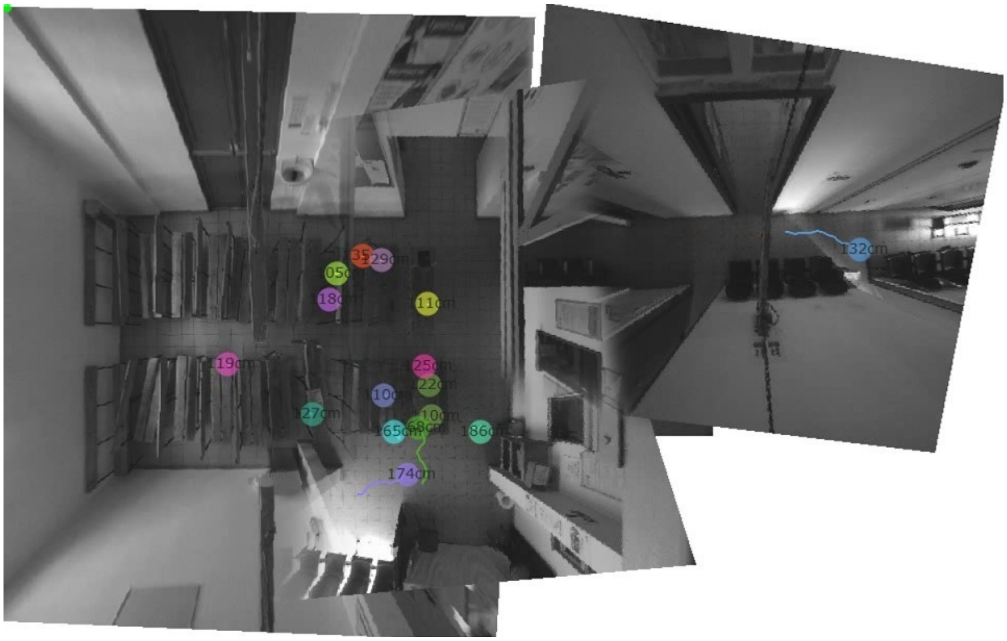

Figure 2

254x160mm (96 x 96 DPI)

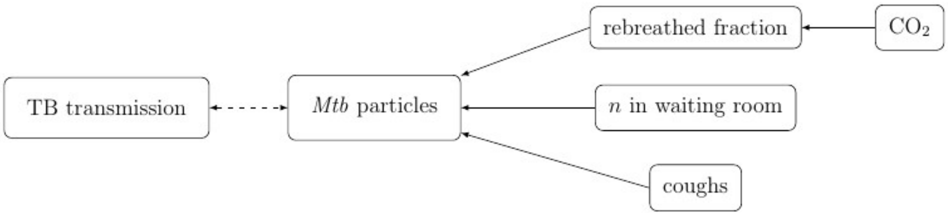

Figure 2

168x53mm (120 x 120 DPI)

UNIVERSITÄT BERN

Medical Faculty

Institute of Social and Preventive Medicine

To the Editor
BMJ Open
Section "Study Protocol"

Bern, December 5, 2019

Re: "*A novel prospective study design to estimate tuberculosis transmission in primary care clinics in African high-burden settings*" – *Study Protocol Section*

Dear Editor,

We are pleased to submit our study protocol in your *Journal*. Tuberculosis (TB) transmission is difficult to measure and its drivers are not well understood. There are still many gaps in our knowledge on TB transmission, particularly the effectiveness of infection control measures at healthcare clinics in high TB and HIV burden settings.

We describe a novel and rapid study design to assess risk factors for airborne transmission at primary care clinics in high TB/HIV burden settings. The study protocol combines a range of different measurements. Using video sensors we will calculate the number of patients, waiting times and anonymous patient movements. In addition, acoustic sound recordings will be used to determine frequency and intensity of patients' coughing. Environmental data will include indoor carbon dioxide levels and relative humidity. We will also extract routinely collected clinical data from electronic databases. The number of *Mycobacterium tuberculosis* particles in the air will be ascertained from dried air filters from bio-aerosol sampling devices and using highly sensitive molecular techniques. The feasibility of our approach was recently demonstrated in a pilot study in a primary care clinic in Cape Town, South Africa, and we are now planning to roll-out a broader study in several countries in sub-Saharan Africa using the described study protocol.

Based on the collected data we will then develop a mathematical model to estimate the risk of TB transmission in these settings as described in the study protocol. The model will be used to estimate the effect of interventions such as separating patient flows or improving ventilation in reducing TB transmission. This will help with logistical and infrastructural planning of primary care clinics to reduce the transmission risk. The results from this broader study have the potential to inform national and international guidelines.

We hope you will find our study protocol is of interest to your readers.

Yours sincerely,

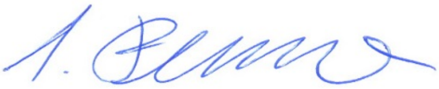

Prof. Lukas Fenner, on behalf of the co-authors

Institute of Social and Preventive Medicine (ISPM), University of Bern
Mittelstrasse 43, CH-3012 Bern, Switzerland
Phone: +41 31 631 35 18 E-Mail: lukas.fenner@ispm.unibe.ch

For peer review only

BMJ Open

A novel prospective study design to estimate tuberculosis transmission in primary care clinics in African high-burden settings

Journal:	*BMJ Open*
Manuscript ID	bmjopen-2019-036214.R1
Article Type:	Protocol
Date Submitted by the Author:	25-Mar-2020
Complete List of Authors:	Zürcher, Kathrin; University of Bern, Institution of Social and Preventive Medicine (ISPM)<br>Morrow, Carl; Desmond Tutu HIV Foundation; University of Cape Town, Institute of Infectious Disease and Molecular Medicine and the Department of Medicine<br>Riou, Julien; University of Bern, Institution of Social and Preventive Medicine (ISPM)<br>Ballif, Marie; University of Bern, Institution of Social and Preventive Medicine (ISPM)<br>Koch, Anastasia; University of Cape Town, Molecular Mycobacteriology Research Unit, Institute of Infectious Disease and Molecular Medicine<br>Bertschinger, Simon; University of Bern, Institution of Social and Preventive Medicine (ISPM); Berne University of Applied Sciences, Medical Informatics<br>Liu, Xin; University of Washington, Paul G. Allen School of Computer Science & Engineering<br>Sharma, Manuja; University of Washington, Paul G. Allen School of Computer Science & Engineering<br>Middelkoop, Keren ; Desmond Tutu HIV Foundation; University of Cape Town, Institute of Infectious Disease and Molecular Medicine<br>Warner, Digby; University of Cape Town, Molecular Mycobacteriology Research Unit, Institute of Infectious Disease and Molecular Medicine<br>Wood, Robin; Desmond Tutu HIV Foundation; University of Cape Town, Institute of Infectious Disease and Molecular Medicine<br>Egger, Matthias; University of Bern, Institution of Social and Preventive Medicine (ISPM); University of Cape Town, Centre for Infectious Disease Epidemiology & Research, School of Public Health & Family Medicine<br>Fenner, Lukas; University of Bern, Institution of Social and Preventive Medicine (ISPM)
**Primary Subject Heading**:	Infectious diseases
Secondary Subject Heading:	Epidemiology, HIV/AIDS
Keywords:	Tuberculosis < INFECTIOUS DISEASES, INFECTIOUS DISEASES, PUBLIC HEALTH, PRIMARY CARE

I, the Submitting Author has the right to grant and does grant on behalf of all authors of the Work (as defined in the below author licence), an exclusive licence and/or a non-exclusive licence for contributions from authors who are: i) UK Crown employees; ii) where BMJ has agreed a CC-BY licence shall apply, and/or iii) in accordance with the terms applicable for US Federal Government officers or employees acting as part of their official duties; on a worldwide, perpetual, irrevocable, royalty-free basis to BMJ Publishing Group Ltd ("BMJ") its licensees and where the relevant Journal is co-owned by BMJ to the co-owners of the Journal, to publish the Work in this journal and any other BMJ products and to exploit all rights, as set out in our licence.

The Submitting Author accepts and understands that any supply made under these terms is made by BMJ to the Submitting Author unless you are acting as an employee on behalf of your employer or a postgraduate student of an affiliated institution which is paying any applicable article publishing charge ("APC") for Open Access articles. Where the Submitting Author wishes to make the Work available on an Open Access basis (and intends to pay the relevant APC), the terms of reuse of such Open Access shall be governed by a Creative Commons licence – details of these licences and which Creative Commons licence will apply to this Work are set out in our licence referred to above.

Other than as permitted in any relevant BMJ Author's Self Archiving Policies, I confirm this Work has not been accepted for publication elsewhere, is not being considered for publication elsewhere and does not duplicate material already published. I confirm all authors consent to publication of this Work and authorise the granting of this licence.

For peer review only

TITLE PAGE *Journal: BMJ Open – Section "Study protocol"*

A novel prospective study design to estimate tuberculosis transmission in primary care clinics in African high-burden settings

Kathrin Zürcher [1], Carl Morrow [2, 3], Julien Riou [1], Marie Ballif [1], Anastasia S. Koch [4], Simon Bertschinger [1, 5], Xin Liu [6], Manuja Sharma [6], Keren Middelkoop [2, 3], Digby F. Warner [4], Robin Wood [2, 3], Matthias Egger [1, 7], Lukas Fenner [1]

1) Institute of Social and Preventive Medicine, University of Bern, Switzerland
2) Desmond Tutu HIV Centre, Mowbray, South Africa
3) Institute of Infectious Disease and Molecular Medicine, University of Cape Town, South Africa
4) Molecular Mycobacteriology Research Unit, Institute of Infectious Disease and Molecular Medicine, University of Cape Town, Cape Town, South Africa.
5) Medical Informatics, Bern University of Applied Sciences, Bern, Switzerland
6) Paul G. Allen School of Computer Science & Engineering, University of Washington, United States
7) Centre for Infectious Disease Epidemiology & Research, School of Public Health & Family Medicine, University of Cape Town, South Africa

Correspondence:

Professor Lukas Fenner, MD
Institute of Social and Preventive Medicine, University of Bern (ISPM)
Mittelstrasse 43, 3012 Bern, Switzerland
Phone: +41 31 631 35 18
E-Mail: lukas.fenner@ispm.unibe.ch

Key words: *Mycobacterium tuberculosis*, Tuberculosis, Transmission, CO_2

Running head: Estimating tuberculosis transmission

Word count, inserts and additional files:

Abstract 305 words, main text 3,130 words

Inserts: 1 table, 3 figures, 37 references

For peer review only - http://bmjopen.bmj.com/site/about/guidelines.xhtml

ABSTRACT

Background: Tuberculosis (TB) transmission is difficult to measure, and its drivers are not well understood. The effectiveness of infection control measures at healthcare clinics and the most appropriate intervention strategies to interrupt transmission are unclear. We propose a novel approach using clinical, environmental and position-tracking data to study the risk of TB transmission at primary care clinics in TB and HIV high burden settings in sub-Saharan Africa.

Methods and analysis: We describe a novel and rapid study design to assess risk factors for airborne TB transmission at primary care clinics in high burden settings. The study protocol combines a range of different measurements. We will collect anonymous data on the number of patients, waiting times and patient movements using video sensors. Also, we will collect acoustic sound recordings to determine the frequency and intensity of coughing. Environmental data will include indoor carbon dioxide levels (CO_2 in parts per million) and relative humidity. We will also extract routinely collected clinical data from the clinic records. The number of *Mycobacterium tuberculosis* particles in the air will be ascertained from dried filter units using highly sensitive digital droplet PCR. We will calculate rebreathed air volume based on people density and CO_2 levels and develop a mathematical model to estimate the risk of TB transmission. The mathematical model can then be used to estimate the effect of possible interventions such as separating patient flows or improving ventilation in reducing transmission. The feasibility of our approach was recently demonstrated in a pilot study in a primary care clinic in Cape Town, South Africa.

Ethics and dissemination: The study was approved by the University of Cape Town (HREC/REF no. 228/2019), the City of Cape Town (ID-8139) and the Ethics Committee of the Canton Bern (2019-02131), Switzerland. The results will be disseminated in international peer-reviewed journals and presented at national and international conferences.

For peer review only - http://bmjopen.bmj.com/site/about/guidelines.xhtml

Strengths and limitations of this study

- Tuberculosis (TB) transmission is difficult to measure, and its drivers are not well understood.
- This protocol describes a novel approach to studying TB transmission using longitudinal study design in primary care clinics in high TB and HIV burden settings.
- This rapid approach will combine a wide range of different measurements, including patient waiting times and movements, acoustic recording of coughing, measurement of carbon dioxide levels and of *Mycobacterium tuberculosis* particles in the air.
- This study will lead to a precise and comprehensive transmission model to measure the effects of various interventions.
- Study limitations pertain to the need of power and stable WiFi over 24 hours for data collection at the primary care clinic, which is often an issue in low- and middle-income countries.

For peer review only

For peer review only - http://bmjopen.bmj.com/site/about/guidelines.xhtml

INTRODUCTION

Tuberculosis (TB), caused by the bacterium *Mycobacterium tuberculosis (Mtb)*, remains a major global public health problem, particularly in the context of HIV and drug resistance. Sub-Saharan Africa is one of the most heavily burdened regions globally, although control measures have been in place since the beginning of the 20th century.[1] Over a century of investment in TB control has reduced TB mortality, but effective strategies are urgently needed to reduce TB transmission.[2] Drivers of the TB epidemic in sub-Saharan Africa are HIV-infection and the resulting immunodeficiency (the strongest risk factor),[1] delayed diagnosis and treatment as well as undetected and untreated cases of TB or drug-resistant TB. These factors allow infectious TB patients to transmit *Mtb* to the community.[3-5] There are still many gaps in our knowledge on TB transmission such as the factors and locations associated with the risk of transmission, the effectiveness of infection control measures at clinics in high-burden settings, and the most appropriate intervention strategies to interrupt transmission.[5-7]

For TB transmission to occur, infected individuals must expel *Mtb* bacilli from their respiratory tract, and an uninfected individual must inhale aerosols containing live bacilli to become infected. Transmission of *Mtb* is difficult to measure due to the lack of an *in vitro* test assay. The preferred approach is to measure presumptive transmission resulting in secondary cases as determined by molecular/genomic epidemiology. TB transmission has traditionally been investigated using contact tracing, analyses of geo-temporal clustering, and molecular typing.[8 9] However, molecular methods require resource-intensive culturing of strains and measure only transmission resulting in secondary cases. Furthermore, contact tracing is difficult to implement in resource-limited settings and may not be an effective control strategy in endemic areas where casual contacts are increasingly recognized as an important contributor to transmission.[10] New approaches are therefore urgently needed.

Mtb is carried in airborne particles (called infectious droplets), which are generated when people with TB cough, sneeze or shout.[5] Indoor carbon dioxide (CO_2) levels can be used to assess the amount of exhaled air in a room, and the amount of rebreathed air.[11-13] Humidity is associated with Mtb survival in the air.[14] Viable *Mtb* particles have been captured from contaminated air.[11 15-17] We describe a unique study design to assess risk factors for airborne TB transmission in primary

For peer review only - http://bmjopen.bmj.com/site/about/guidelines.xhtml

care clinics in high TB/HIV burden settings. The approach combines a range of relevant measurements, including data on patient and infrastructure, movements of patients through the facility, coughing, environmental indoor CO_2 levels, and concentration of *Mtb* particles in the air.

We hypothesize that (i) exposure to *Mtb* particles at the clinic can be estimated by studying the patient flow and CO_2 levels; (ii) the number of individuals present, the rebreathed fraction and the frequency of coughs influences exposure to *Mtb*; (iii) clinics with small and crowded waiting rooms, low ventilation, and suboptimal patient separation have an increased risk of TB transmission.

METHODS

Study design

Longitudinal study with data collection at the levels of the patients, the clinic, and the environment. Data collection will take place over four weeks. Figure 1 shows the floor plan of an exemplary primary clinic with the planned study activities, including the recording of patient movements coughing, CO_2 levels and *Mtb* particles in the air.

Study setting and study population

The study will take place at several ART or primary care clinics in countries participating in the International Epidemiology Database to Evaluate AIDS in Southern Africa (IeDEA-SA) collaboration with a high TB and HIV burden. These clinics are located in urban and peri-urban communities with predominantly young and black African residents. TB and HIV are both prevalent in these communities.

Patient-level variables

Video sensor data

A person-tracking sensor system developed by Xovis (Zollikofen, Switzerland and Cambridge, MA; see www.xovis.com) will be used to monitor the clinic attendees' movements. The data will be used to calculate waiting times, the number of people in different locations, and the average distance between people and to identify highly frequented areas. Several sensors will be installed to cover the clinic area, calibrated and validated. Sensors with overlapping ranges will be combined for the seamless

For peer review only - http://bmjopen.bmj.com/site/about/guidelines.xhtml

coverage of people's movements over large areas (Figure 2). The raw data consist of the person's height, time, date, and the position (x-y coordinates) for each unique individual during the duration of their stay within the clinic. The data are captured every 0.25 seconds. The raw data are then parsed by a python script to calculate the height, total movement, and observation time at different locations, and to visualize hotspots.

The sensors have four levels of privacy. In our study, privacy will be set to level 2, which means that the data are a fully anonymized stream of the coordinates of moving dots. These dots will be linked probabilistically to the anonymized clinical data thus excluding any risk of re-identification. Further, the images of tracked individuals taken by the sensors will not be stored (see also the data privacy and security statement by Xovis).[18 19]

Patient and clinic data

We will extract clinical data from the electronic patient registry for all patients who visited the clinics during the four weeks of data collection. The data will include date and time of registration, age, sex, height (used for linkage with video sensor data), HIV status, presumptive TB ("TB suspects"), mode of TB diagnosis (sputum smear microscopy, Xpert MTB/RIF, mycobacterium culture), TB diagnostic test results, date of TB treatment start, current anti-TB treatment regimen. We will not extract any personal data, such as names or social security numbers. The clinic-level data will include information such as the setting (urban or rural), level of care, the number of patients seen each year, availability of adult and paediatric care, TB control measures (natural ventilation, use of masks, separation of TB patients or coughers), and the floor plan of the clinics. The data will be collected using the web-based REDCap data entry system (www.redcap.org).[20]

Definitions

Presumptive TB refers to a patient who presents with clinical symptoms or signs suggestive of TB. A bacteriologically confirmed TB case is a person for whom a biological specimen is positive by smear, microscopy, culture or rapid diagnostic such as Xpert MTB/RIF or line probe assay (LPA).[21]

Environmental variables

CO_2, relative humidity and temperature monitoring

For peer review only - http://bmjopen.bmj.com/site/about/guidelines.xhtml

We will use CO_2 monitors (Digital CO2 Monitor Carbon Dioxide Meter XE-2000, XEAST, Guangdong, China) which include the COZIR-A sensor for ambient CO_2 levels of 0–1% (Gas Sensing Solutions, Cumbernauld, Scotland). The monitors will record indoor CO_2 concentrations (in parts per million [ppm]), temperature, and relative humidity (RH) at minute intervals. Data from the five monitors are stored and exported in serial digital format.[15 16] At each clinic we will install five monitors to cover the most visited spaces and a control area outside the clinic (Figure 1). The monitors auto-calibrate over time to a standard minimum value of 400 ppm. This value is close to the average monthly outdoor carbon dioxide concentration measured at Cape Point, South Africa (405.48 ppm in December 2018).[22] Monitors will be run for one week before the start of the study to allow them to calibrate and to confirm that they delivered comparable data.

Cough monitoring

We will install a microphone (RØDE NT-USB, Sydney, Australia) near the ceiling to continuously record the sounds in the waiting room (Figure 1). *CoughSense*, a deep learning cough detection algorithm based on MXNet, an open-source deep-learning software framework, was developed to classify audio signals as coughing or other sounds.[23] The algorithm uses spectrograms extracted from the raw audio for classification. The model was trained and tested using multiple audio recordings obtained through clinical and ambulatory deployments. We will identify audio records with cough sounds and calculate the cough frequency, intensity and duration. The cough data will be linked with the CO_2 data and video senor data by time and date. Finally, we will probabilistically link the video sensor data (i.e. the moving dots) with the clinical data by the height of the patient, time and date of the visit.

Bio-aerosol sampling and molecular detection

We will collect *Mtb* particles from the air using mobile bio-aerosol sampling devices (Dry Filter Unit [DFU]-1000, Lockheed Martin Integrated Systems, Gaithersburg, MD, USA). The DFU 1000 is a portable biological air sampler. Ambient air is drawn through 1 µm polyester-felt filters at a rate of ~1000 l/min via an electrical blower. The DFU is a useful collection system for low concentration aerosols, capturing particles of 1 µm or larger, and allowing easy access for the retrieval of filters. One DFU will be placed in the waiting room and the other in the TB treatment room (Figure 1).

For peer review only

Each DFU collects air for 7 hours (two periods of 3.5 hours) every day onto two filters. Due to logistical and human resource limitations, we will be able to change filters only twice per day. Therefore, we decided to run the mobile bio-aerosol sampling devices during the busiest time at the clinic, between 7 am and 2 pm. All other data will be collected from 7 am to 4 pm.

As previously described,[17] duplicate filters from each sampling session will be transferred to 50mL Falcon tubes and vortexed in sterile phosphate buffered saline (PBS) (with 0.05% Tween 80). Following centrifugation at 3750 rpm for 15 mins, filters will be removed and the pellet subjected to DNA extraction. DNA from *Mtb* cells will be extracted using an in-house lysis buffer with subsequent pelleting (centrifugation 13000 rpm for 10 mins) of DNA and resuspension in 50 µl of Tris-EDTA buffer (10mM Trist, 1mM EDTA, pH 8.0). Given that ddPCR is relatively robust against inhibitors, no further DNA purification will be required. The primer/probe combinations and reaction conditions for *Mtb* specific ddPCR have been described.[16] Samples with known amounts of purified *Mtb* DNA (0.01ng and 0.001ng) will be included to serve as positive and nuclease-free water as negative control. The data generated from the ddPCR reaction will be analyzed via the Umbrella pipeline, [24] using wells with a minimum of 10,000 droplets.

Statistical analyses

We will describe the data and examine associations between sources of data and then use results to parameterize a mathematical model (Table 1). The statistical analysis will quantify the joint association between the clinical and environmental variables and the number of *Mtb* particles measured by the mobile aerosol sampling. To this end, we will use Poisson regression, with the number of *Mtb* particles by the period of time as the dependent variable, considered as a proxy measure for the risk of TB transmission. As this variable is measured by periods of 3.5 hours, the other variables will be aggregated over the same periods. We will consider all combinations of independent variables, including second-order interactions, and compare the model using standard model selection methods. We will use variable selection methods in a Bayesian framework, including the deviance information criterion (DIC) and the leave-one-out information criterion (LOOIC).[25]

We will operationalize the variables as follows:

For peer review only - http://bmjopen.bmj.com/site/about/guidelines.xhtml

1. *Patient data*: Numbers and characteristics of patients consulting the clinic overall will be summarized using descriptive statistics.
2. *Video sensor data:* Raw data about indiviudals movements will be transformed into waiting times until medical consultation, number of individuals in the different locations, highly frequented areas in the clinic and the average distance between patients (i.e. clustering). We will link each tracked individual to the clinical data collected from the clinic's database (i.e. TB/HIV diagnosis), using the order of arrival and time of registration.
3. *CO_2 data*: Measurements of CO_2 concentration at the different locations in the clinic (waiting room, registration desk, TB treatment room), together with estimates in outdoor air, will be used to estimate the proportion of air in the different locations that was expired by individuals, the *rebreathed fraction*.[12] We will use a modified Wells-Riley model appropriate for non-steady states conditions of ventilation and number of individuals to describe and calculate the shared rebreathed air in the different locations in either liter/minute or liter/day and the air exchange (liter/hour per person). Table 2 describes the parameters to calculate the shared rebreathed air and the air exchange.[13 16 26 27]

The proportion of rebreathed air (*f*) will be calculated from the excess CO_2 measured indoors, divided by the exhaled CO_2 *(Ca)*:

Equation 1:

$$f = \frac{(C - Co)}{Ca}$$

(see Table 1 for definitions of parameters in this and the following equations).

Before we can calculate the rebreathed air volume (RAV) we will need the rebreathed proportion from other people (fo). Therefore we need to know the number of people *(n)* present at each time point at the clinic:

Equation 2:

$$fo = f \times \frac{(n-1)}{n}$$

Finally, we can calculate the RAV for each minute by multiplying fo and the minute respiratory volume (8 liters per minute, *[p]*) as:

For peer review only

For peer review only - http://bmjopen.bmj.com/site/about/guidelines.xhtml

Equation 3

$$Rebreathed\ air\ volume\ (RAV) = (pfo)$$

In the next step, we aim to calculate the ventilation rate (air exchange). The indoor CO_2 generated rate is the product of the average volume of gas exhaled per person (0.13 l/s per person*, [V]*) and the CO_2 concentration of the exhaled air:

Equation 4:

$$Indoor\ CO_2\ generation\ rate\ in\ l/s\ per\ person\ (G) = VxCa$$

The ventilation rate is expressed as:

Equation 5:

$$Ventilation\ rate\ in\ l/s\ per\ person\ (Q) = \frac{G}{C - Co}$$

To calculate the air exchange per hour (ACH, *Equation 6*) we need to know the volume of air in a given location.

4. *Relative humidity and temperature:* We will describe changes in relative humidity and temperature over the day.
5. *Cough sounds*: Frequency, intensity, duration of recorded coughs per time period.[28 29]
6. *Detection of Mtb in bio-aerosol sampling*: The number of *Mtb* genome copies present in each sample.

Mathematical modelling

A mathematical model (Figure 3) will integrate all sources of data to model the risk of TB transmission and identify key drivers of transmission.[13 26 30 31] We hypothesize that the number of individuals present, the rebreathed fraction and the frequency of coughs will have the greatest influence on the risk. As we do not observe transmission events, we will use *yu*, the number of *Mtb* genomes counted by the bio-aerosol sampling for each 6-hour period *u*, as the dependent variable. The model will describe *yu* as a Poisson process with time-dependent intensity *λt* (also called a Cox process). This intensity can be interpreted as a proxy for the (unobserved) risk of TB transmission. We will model *λtu* using 10-minutes time periods *t* within *u*. In a first simple model, *λtu*

will integrate multiple independent variables *xtu*: the number of individuals in the waiting room during time *t*, the rebreathed fraction and the number of coughs. The model will be estimated in a Bayesian framework using *Stan*, a probabilistic programming language.[32]

We will use several metrics to assess the goodness of fit of the model. We will then add more complexity to the model by integrating other independent variables such as the characteristics of the patients, shared rebreathed air (CO_2 monitors),[12 13 15 16] patient flow (clustering of individuals, movements of patients), and other potential drivers (intensity/frequency/duration of coughs).[28 29]

Having developed the model of the risk of TB transmission, we will evaluate the possible effect of interventions such as limiting the number of patients in the waiting room, separating coughers or increasing ventilation. All analyses will be performed in R (version 3.6.0) or STATA (version 15.1, Stata Corporation, Texas, USA).

Reporting

The results from this study will be reported following the recommendations of the STROBE statement.[33]

Pilot study

We conducted a pilot study in Cape Town (South Africa) to examine the feasibility of our approach (Figure 1). The pilot study took place at a primary care clinic, which incorporates HIV counselling and testing, and a TB clinic for the diagnosis and management of TB. The clinic is situated in Masiphumelele, a large settlement of formal and semi-formal housing in Cape Town, which has previously been described.[34 35] The clinic is open on workdays from Monday till Friday from 7 am till 4 pm (opening hours of the clinic). We studied clinic activities over four weeks on workdays between July 25 and August 23 2019.

Data collection was successful overall, however, we experienced several power cuts during the pilot study. An important lesson learned is that power banks for laptops and WiFi routers are needed, as well as UPS power stabilizers and access to a generator as back-up for the DFU and CO_2 monitors.

Ethics statement

For peer review only - http://bmjopen.bmj.com/site/about/guidelines.xhtml

The University of Cape Town Faculty of Health Sciences Human Research Ethics Committee (HREC/REF: 228/2019), the City of Cape Town (Project ID 8139) and the Ethics Committee of the Canton of Bern (2019-02131), Switzerland approved the pilot study. Expansion of the study will include other clinics in the greater Cape Town metropolitan area, but also clinics in Zambia, Zimbabwe or Malawi, including urban and rural sites to ensure representative and generalizable results. Separate ethical approval will be sought from the relevant ethics committees or institutional review boards.

DISCUSSION

TB control is particularly relevant for sub-Saharan Africa, which carries a disproportionally large portion of the global burden of both TB and HIV. There is an urgent need to understand the drivers of TB transmission to reduce TB incidence using new intervention approaches.[1] A better understanding of transmission, coupled with a rapid test system are likely to contribute to improving TB control in clinic settings. This project will provide new insights into the complex TB transmission framework at a primary care clinic in an endemic setting using clinical data, CO_2 levels, cough analyses, video tracking and *Mtb* particles sampled from the air. Although the large-scale implementation and evaluation of interventions are beyond the scope of this study, the results will generate new hypotheses and opportunities for public health intervention studies (e.g., randomized controlled or cluster-randomized trials).

Importantly, with mathematical models based on real-life data, we can evaluate the likely effect of interventions thus improving intervention studies, and inform help with logistical and infrastructural planning of primary care clinics to reduce the transmission risk. Having established feasibility in one clinic in South Africa, we are planning to collect data in several countries in sub-Saharan Africa. The results from this broader study have the potential to inform national and international guidelines to reduce TB transmission at health care centres.

Strengths and limitations: This is a novel and rapid approach to studying TB transmission combining a wide range of different measurements, which goes beyond

For peer review only

For peer review only - http://bmjopen.bmj.com/site/about/guidelines.xhtml

the traditional methods such as contact tracing, geo-temporal clustering or molecular genotyping. It will lead to a comprehensive transmission model to measure the effects of various interventions in the clinic setting, paving the way for future studies. Study limitations pertain to the need for stable electrical power and WiFi over 24 hours for data collection at the primary care clinic, which is often an issue in low- and middle-income countries. We will address this problem by using UPS power stabilizers with access to generator back-up power.

PATIENT AND PUBLIC INVOLVEMEMT

We discussed the aims and design study with local clinic staff, colleagues at the University of Cape Town and public health specialists early on in the planning phase and developed the specific objectives and data collection procedures in collaboration with them. Patients were not involved in the design, recruitment or conduct of the study. We will make the results of this study available to the participating clinics and the public health authorities.

DISSEMINATION

The results will be disseminated in international peer-reviewed journals and presented at national and international conferences.

AUTHORS CONTRIBUTIONS

Overall concept: KZ, CM, MB, RW, ME, LF. Medical informatics: SB, JR. Cough sound concept: XL, MS, CM, RW. Video sensor concept: KZ, JB, SB, ME, LF. Patient data concept: CM, KM, RW. Laboratory work concept: CM, AK, KM, DW, RW. Modelling work concept: KZ, JR, SB, ME, LF. Pilot study coordination: CM, KM, RW. KZ, MB, ME, LF wrote the first draft of the paper, which was reviewed by all authors and revised on the basis of the comments received by co-authors. All authors approved the final version of the manuscript.

ACKNOWLEDGEMENTS

For peer review only - http://bmjopen.bmj.com/site/about/guidelines.xhtml

We would like to thank Mbali Mohlamonyane for setting up the bio-aerosol sample collection system, Zeenat Hoosen and Ronnett Seldon for developing the laboratory protocols. We are also grateful to the clinical manager,the staff members at the clinic and all patients who contributed to this study. Finally, we would like to thank for the support from the City of Cape Town for the use of one of their clinic facilities. Research findings and recommendations do not represent an official view of the City of Cape Town.

SOURCES OF SUPPORT

The roll-out of this study is supported by a grant from the Swiss National Science Foundation (grant no. CRSK-3_190781). KZ and LF are supported by the National Institute of Allergy and Infectious Diseases (NIAID) through grant no. 5U01-AI069924-05, ME by special project funding (grant no. 174281) from the Swiss National Science Foundation.

COMPETING INTERESTS

All authors declare that they have no conflicts of interest.

For peer review only - http://bmjopen.bmj.com/site/about/guidelines.xhtml

REFERENCES

1. World Health Organization. Global tuberculosis report 2018. *World Health Organization Document* 2018;WHO/HTM/TB/2018:277.
2. Hermans S, Horsburgh CR, Jr., Wood R. A Century of Tuberculosis Epidemiology in the Northern and Southern Hemisphere: The Differential Impact of Control Interventions. *PloS one* 2015;10(8):e0135179. doi: 10.1371/journal.pone.
3. Wood R, Lawn SD, Caldwell J, et al. Burden of new and recurrent tuberculosis in a major South African city stratified by age and HIV-status. *PloS one* 2011;6(10):e25098. doi: 10.1371/journal.pone.0025098
4. Anonymous. Tuberculosis: a much-needed focus on transmission. *Lancet Respir Med* 2019;7(6):465. doi: 10.1016/S2213-2600(19)30148-1
5. Rieder HL. Epidemiologic basis of tuberculosis control. In: International Union Against Tuberculosis and Lung Disease, ed. 1 ed. Paris: International Union Against Tuberculosis and Lung Disease, 1999:1-162.
6. Lin HH, Dowdy D, Dye C, et al. The impact of new tuberculosis diagnostics on transmission: why context matters. *Bull World Health Organ* 2012;90:739-47A.
7. Middelkoop K, Mathema B, Myer L, et al. Transmission of tuberculosis in a South African community with a high prevalence of HIV infection. *J Infect Dis* 2015;211:53-61.
8. Walker TM, Ip CL, Harrell RH, et al. Whole-genome sequencing to delineate *Mycobacterium tuberculosis* outbreaks: a retrospective observational study. *The Lancet Infect. Dis* 2012 doi: S1473-3099(12)70277-3 [pii];10.1016/S1473-3099(12)70277-3 [doi]
9. Cook VJ, Shah L, Gardy J, et al. Recommendations on modern contact investigation methods for enhancing tuberculosis control. *IJTLD* 2012;16:297-305.
10. Middelkoop K, Bekker LG, Morrow C, et al. Decreasing household contribution to TB transmission with age: a retrospective geographic analysis of young people in a South African township. *BMC Infect Dis* 2014;14:221. doi: 10.1186/1471-2334-14-221
11. Andrews JR, Morrow C, Walensky RP, et al. Integrating social contact and environmental data in evaluating tuberculosis transmission in a South African township. *J Infect Dis* 2014 doi: 10.1093/infdis/jiu138

For peer review only

For peer review only - http://bmjopen.bmj.com/site/about/guidelines.xhtml

12. Myatt TA, Johnston SL, Rudnick S, et al. Airborne rhinovirus detection and effect of ultraviolet irradiation on detection by a semi-nested RT-PCR assay. *BMC public health* 2003;3:5.

13. Rudnick SN, Milton DK. Risk of indoor airborne infection transmission estimated from carbon dioxide concentration. *Indoor Air* 2003;13(3):237-45.

14. Sornboot J, Aekplakorn W, Ramasoota P, et al. Detection of airborne Mycobacterium tuberculosis complex in high-risk areas of health care facilities in Thailand. *IJTLD* 2019;23(4):465-73. doi: 10.5588/ijtld.18.0218

15. Wood R, Morrow C, Ginsberg S, et al. Quantification of shared air: a social and environmental determinant of airborne disease transmission. *PloS one* 2014;9(9):e106622. doi: 10.1371/journal.pone.0106622

16. Richardson ET, Morrow CD, Kalil DB, et al. Shared air: a renewed focus on ventilation for the prevention of tuberculosis transmission. *PloS one* 2014;9(5):e96334. doi: 10.1371/journal.pone.0096334

17. Patterson B, Morrow C, Singh V, et al. Detection of Mycobacterium tuberculosis bacilli in bio-aerosols from untreated TB patients. *Gates Open Res* 2017;1:11. doi: 10.12688/gatesopenres.12758.1

18. Xovis. Guaranteed data privacy Zollikofen2018 [Available from: https://www.xovis.com/technology/detail/guaranteed-data-privacy/ accessed 03.12.2019 2019.

19. Xovis. Xovis' Privacy Policy Zollikofen2018 [Available from: https://www.xovis.com/data-privacy/ accessed 03.12.2019 2019.

20. Harris PA, Taylor R, Minor BL, et al. The REDCap consortium: Building an international community of software platform partners. *J. Biomed. Inform* 2019;95:103208. doi: 10.1016/j.jbi.2019.103208

21. World Health Organization. Definitions and reporting framework for tuberculosis–2013 revision: World Health Organization 2013.

22. National Oceanic & Atmospheric Administration. Earth System Research Laboratory; Global Monitoring Division [updated 09.11.2019. Available from: https://www.esrl.noaa.gov/gmd/dv/data/index.php?site=CPT&parameter_name=Carbon%2BDioxide,%20accessed%2012%20November%202019 accessed 12.11.2019 2019.

23. Chen T, Li M, Li Y, et al. Mxnet: A flexible and efficient machine learning library for heterogeneous distributed systems. *arXiv preprint arXiv:151201274* 2015

For peer review only - http://bmjopen.bmj.com/site/about/guidelines.xhtml

24. Jacobs BKM, Goetghebeur E, Vandesompele J, et al. Model-Based Classification for Digital PCR: Your Umbrella for Rain. *Anal. Chem* 2017;89(8):4461-67. doi: 10.1021/acs.analchem.6b04208

25. Vehtari A, Gelman A, Gabry J. Practical Bayesian model evaluation using leave-one-out cross-validation and WAIC. *Stat. Comput* 2017;27(5):1413-32.

26. Riley RL, Mills CC, Nyka W, et al. Aerial dissemination of pulmonary tuberculosis. A two-year study of contagion in a tuberculosis ward. 1959. *Am J Epidemiol* 1995;142(1):3-14. doi: 10.1093/oxfordjournals.aje.a117542

27. Wells WF. On air-borne infection*: Study II.droplets and droplet nuclei. *Am J Epidemiol* 1934;20(3):611-18. doi: 10.1093/oxfordjournals.aje.a118097

28. Birring SS, Fleming T, Matos S, et al. The Leicester Cough Monitor: preliminary validation of an automated cough detection system in chronic cough. *Eur Respir J* 2008;31(5):1013-8. doi: 10.1183/09031936.00057407

29. Botha GHR, Theron G, Warren RM, et al. Detection of tuberculosis by automatic cough sound analysis. *Physiol Meas* 2018;39(4):045005. doi: 10.1088/1361-6579/aab6d0

30. Gammaitoni L, Nucci MC. Using a mathematical model to evaluate the efficacy of TB control measures. *Emerg Infect Dis* 1997;3(3):335-42. doi: 10.3201/eid0303.970310

31. Issarow CM, Mulder N, Wood R. Modelling the risk of airborne infectious disease using exhaled air. *J. Theor. Biol* 2015;372(0):100-06. doi: http://dx.doi.org/10.1016/j.jtbi.2015.02.010

32. Carpenter J, Crutchley P, Zilca RD, et al. Correction: Seeing the "Big" Picture: Big Data Methods for Exploring Relationships Between Usage, Language, and Outcome in Internet Intervention Data. *J Med Internet Res* 2017;19(12):e347. doi: 10.2196/jmir.8099

33. Vandenbroucke JP, von Elm E, Altman DG, et al. Strengthening the Reporting of Observational Studies in Epidemiology (STROBE): Explanation and Elaboration. *PLOS Med* 2007;4(10):e297. doi: 10.1371/journal.pmed.0040297

34. Wood R, Middelkoop K, Myer L, et al. Undiagnosed tuberculosis in a community with high HIV prevalence: implications for tuberculosis control. *Am J Respir Crit Care Med* 2007;175(1):87-93.

For peer review only

For peer review only - http://bmjopen.bmj.com/site/about/guidelines.xhtml

35. Middelkoop K, Bekker LG, Myer L, et al. Antiretroviral therapy and TB notification rates in a high HIV prevalence South African community. *J. Acquir. Immune Defic. Syndr (1999)* 2011;56(3):263-9. doi: 10.1097/QAI.0b013e31820413b3

36. Fernstrom A, Goldblatt M. Aerobiology and its role in the transmission of infectious diseases. *J Pathog* 2013;2013:493960. doi: 10.1155/2013/493960

37. Emmerich SJ, Persily AK. State-of-the-art review of CO2 demand controlled ventilation technology and application: Citeseer 2001.

For peer review only

TABLES AND FIGURE

Table 1: Description of the measurements

Data source	Parameter	Description	Unit	Measurement taken by:
CO_2 monitor	CO_2	Observed CO_2 concentration in the indoor air per minute and a control in the outdoor air. Based on CO_2 levels (parts per million [ppm]) and people density, we will calculate rebreathed air volume (RAV), which is used as a proxy for airborne TB transmission.[16]	ppm	minute and date
	Relative humidity	Data on the effects of relative humidity on the survival of airborne bacteria are inconsistent.[27 36] However, a recent study found that relative humidity above 65% is associated with *Mtb* survival in the air.[14]	%	minute and date
	Temperature	Temperatures above 24°C are required to reduce airborne bacteria survival.[27 36]	°C	minute and date
Cough recording	Frequency	One of the typical symptoms of TB is coughing; coughing is also the main way of transmission.	n	minute or day and date
	Duration	Duration of each cough is different from healthy and people with TB or other lung diseases [29]	sec	cough by minute and date
	Intensity	Intensity of each cough is different from healthy and people with TB or other lung diseases. [29]	decibel	cough by minute and date
Mobile aerosol sampling	*Mtb* DNA copies	Detection of *Mtb* particles in the air by filter or per day (7am to 2pm).[17]	Copies per microliters	filter (ca.3.5h sampling) or per day
Video sensor	Number of people	From the raw data (x-y coordinates) we can calculate the number of people at a given location by 0.25 seconds and by minute.	n of people	0.25 seconds or minute and by date
	Time spent at a given location	From the raw data (x-y coordinates) we can calculate for each person their time spent at different locations.	minutes	minute and date
Patient charts	Number of registered patients	All patients who are visiting the clinic are registered.	n of registered patient	minute and day
	Number of presumptive TB and of TB patients	From all registered patients we will know the number of presumptive TB and of TB patients.	n of presumptive TB and TB patients	minute and day

For peer review only

For peer review only - http://bmjopen.bmj.com/site/about/guidelines.xhtml

553 **Table 2: Description of the variables to calculate the shared rebreathed air**
554 **volume and air exchange as well as the parameters to construct the**
555 **mathematical transmission model.**
556

Parameter	Description	Value
C	Observed CO_2 concentration in the indoor air per minute	observed
Co	CO_2 concentration in the outdoor air per minute	400-420ppm[22]
Ca	CO_2 concentration in the exhaled air	38000-40000ppm[13 37]
f	Proportion of rebreathed air	equation
n	Number of people recorded at the location	observed
fo	Rebreathed proportion from other people	equation
p	Minute respiratory volume	8 l/per minutes[37]
RAV	Rebreathed air volume	equation
V	Average volume of gas exhaled per person	0.13 l/s per person[16]
G	Indoor CO_2 generation rate (l/s per person)	equation
Q	Ventilation rate (l/s per person)	equation
vol	Volume of the room	calculated

557
558
559

For peer review only - http://bmjopen.bmj.com/site/about/guidelines.xhtml

Figure 1: Floor plan of the pilot primary care clinic in Cape Town, South Africa, indicating study measurements.

Figure 2: Output from videao sensors with moving dots showing the tracked persons. The numbers in the dots indicate the height of patients. The different sectors covered by the sensors are merged. Images from the pilot study.

Figure 3: Model structure.

For peer review only

For peer review only - http://bmjopen.bmj.com/site/about/guidelines.xhtml

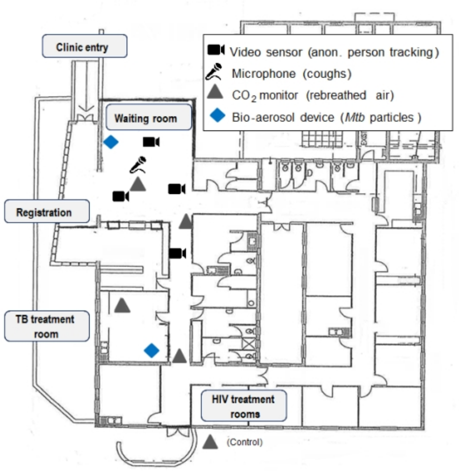

Figure 1: Floor plan of the pilot primary care clinic in Cape Town, South Africa, indicating study measurements

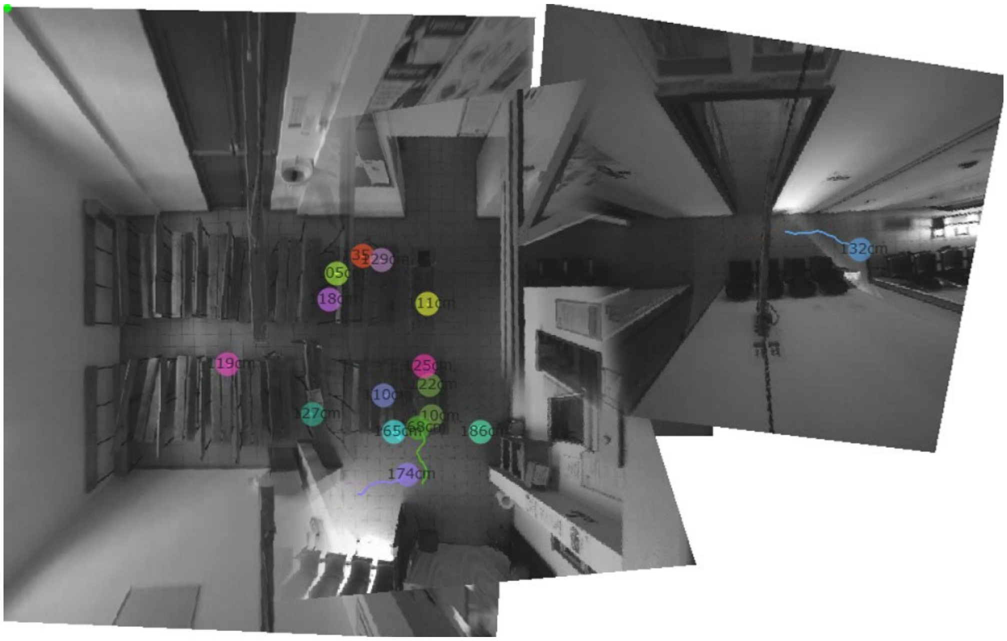

Figure 2: Output from videao sensors with moving dots showing the tracked persons. The numbers in the dots indicate the height of patients. The different sectors covered by the sensors are merged. Images from the pilot study.

81x51mm (300 x 300 DPI)

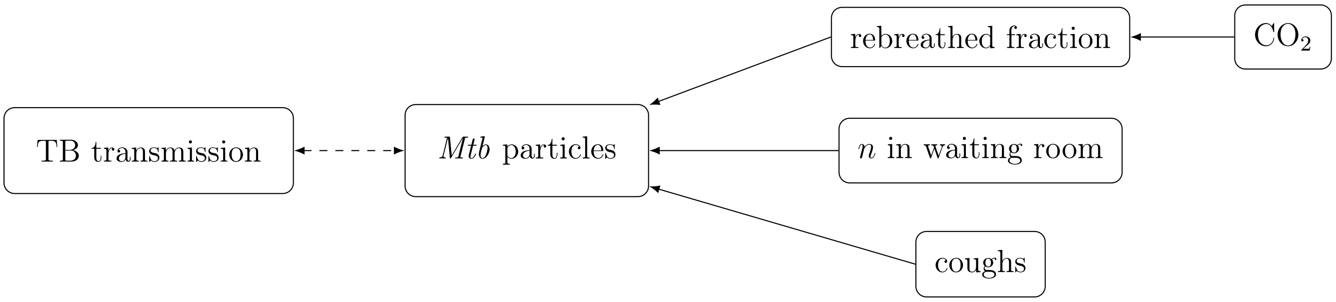

For peer review only

BMJ Open

Novel approach to estimate tuberculosis transmission in primary care clinics in sub-Saharan Africa: protocol of a prospective study

Journal:	*BMJ Open*
Manuscript ID	bmjopen-2019-036214.R2
Article Type:	Protocol
Date Submitted by the Author:	27-Apr-2020

Complete List of Authors:	Zürcher, Kathrin; University of Bern, Institution of Social and Preventive Medicine (ISPM)<br>Morrow, Carl; Desmond Tutu HIV Foundation; University of Cape Town, Institute of Infectious Disease and Molecular Medicine and the Department of Medicine<br>Riou, Julien; University of Bern, Institution of Social and Preventive Medicine (ISPM)<br>Ballif, Marie; University of Bern, Institution of Social and Preventive Medicine (ISPM)<br>Koch, Anastasia; University of Cape Town, Molecular Mycobacteriology Research Unit, Institute of Infectious Disease and Molecular Medicine<br>Bertschinger, Simon; University of Bern, Institution of Social and Preventive Medicine (ISPM); Berne University of Applied Sciences, Medical Informatics<br>Liu, Xin; University of Washington, Paul G. Allen School of Computer Science & Engineering<br>Sharma, Manuja; University of Washington, Paul G. Allen School of Computer Science & Engineering<br>Middelkoop, Keren ; Desmond Tutu HIV Foundation; University of Cape Town, Institute of Infectious Disease and Molecular Medicine<br>Warner, Digby; University of Cape Town, Molecular Mycobacteriology Research Unit, Institute of Infectious Disease and Molecular Medicine<br>Wood, Robin; Desmond Tutu HIV Foundation; University of Cape Town, Institute of Infectious Disease and Molecular Medicine<br>Egger, Matthias; University of Bern, Institution of Social and Preventive Medicine (ISPM); University of Cape Town, Centre for Infectious Disease Epidemiology & Research, School of Public Health & Family Medicine<br>Fenner, Lukas; University of Bern, Institution of Social and Preventive Medicine (ISPM)

**Primary Subject Heading**:	Infectious diseases
Secondary Subject Heading:	Epidemiology, HIV/AIDS
Keywords:	Tuberculosis < INFECTIOUS DISEASES, INFECTIOUS DISEASES, PUBLIC HEALTH, PRIMARY CARE

For peer review only - http://bmjopen.bmj.com/site/about/guidelines.xhtml

I, the Submitting Author has the right to grant and does grant on behalf of all authors of the Work (as defined in the below author licence), an exclusive licence and/or a non-exclusive licence for contributions from authors who are: i) UK Crown employees; ii) where BMJ has agreed a CC-BY licence shall apply, and/or iii) in accordance with the terms applicable for US Federal Government officers or employees acting as part of their official duties; on a worldwide, perpetual, irrevocable, royalty-free basis to BMJ Publishing Group Ltd ("BMJ") its licensees and where the relevant Journal is co-owned by BMJ to the co-owners of the Journal, to publish the Work in this journal and any other BMJ products and to exploit all rights, as set out in our licence.

The Submitting Author accepts and understands that any supply made under these terms is made by BMJ to the Submitting Author unless you are acting as an employee on behalf of your employer or a postgraduate student of an affiliated institution which is paying any applicable article publishing charge ("APC") for Open Access articles. Where the Submitting Author wishes to make the Work available on an Open Access basis (and intends to pay the relevant APC), the terms of reuse of such Open Access shall be governed by a Creative Commons licence – details of these licences and which Creative Commons licence will apply to this Work are set out in our licence referred to above.

Other than as permitted in any relevant BMJ Author's Self Archiving Policies, I confirm this Work has not been accepted for publication elsewhere, is not being considered for publication elsewhere and does not duplicate material already published. I confirm all authors consent to publication of this Work and authorise the granting of this licence.

For peer review only

TITLE PAGE *Journal: BMJ Open – Section "Study protocol"*

Novel approach to estimate tuberculosis transmission in primary care clinics in sub-Saharan Africa: protocol of a prospective study

Kathrin Zürcher [1], Carl Morrow [2, 3], Julien Riou [1], Marie Ballif [1], Anastasia S. Koch [4], Simon Bertschinger [1, 5], Xin Liu [6], Manuja Sharma [6], Keren Middelkoop [2, 3], Digby F. Warner [4], Robin Wood [2, 3], Matthias Egger [1, 7], Lukas Fenner [1]

1) Institute of Social and Preventive Medicine, University of Bern, Switzerland
2) Desmond Tutu HIV Centre, Mowbray, South Africa
3) Institute of Infectious Disease and Molecular Medicine, University of Cape Town, South Africa
4) Molecular Mycobacteriology Research Unit, Institute of Infectious Disease and Molecular Medicine, University of Cape Town, Cape Town, South Africa.
5) Medical Informatics, Bern University of Applied Sciences, Bern, Switzerland
6) Paul G. Allen School of Computer Science & Engineering, University of Washington, United States
7) Centre for Infectious Disease Epidemiology & Research, School of Public Health & Family Medicine, University of Cape Town, South Africa

Correspondence:

Professor Lukas Fenner, MD
Institute of Social and Preventive Medicine, University of Bern (ISPM)
Mittelstrasse 43, 3012 Bern, Switzerland
Phone: +41 31 631 35 18
E-Mail: lukas.fenner@ispm.unibe.ch

Key words: *Mycobacterium tuberculosis*, Tuberculosis, Transmission, CO_2

Running head: Estimating tuberculosis transmission

Word count, inserts and additional files:

Abstract 305 words, main text 3,130 words

Inserts: 1 table, 3 figures, 37 references

For peer review only

ABSTRACT

Introduction: Tuberculosis (TB) transmission is difficult to measure, and its drivers are not well understood. The effectiveness of infection control measures at healthcare clinics and the most appropriate intervention strategies to interrupt transmission are unclear. We propose a novel approach using clinical, environmental and position-tracking data to study the risk of TB transmission at primary care clinics in TB and HIV high burden settings in sub-Saharan Africa.

Methods and analysis: We describe a novel and rapid study design to assess risk factors for airborne TB transmission at primary care clinics in high burden settings. The study protocol combines a range of different measurements. We will collect anonymous data on the number of patients, waiting times and patient movements using video sensors. Also, we will collect acoustic sound recordings to determine the frequency and intensity of coughing. Environmental data will include indoor carbon dioxide levels (CO_2 in parts per million) and relative humidity. We will also extract routinely collected clinical data from the clinic records. The number of *Mycobacterium tuberculosis* particles in the air will be ascertained from dried filter units using highly sensitive digital droplet PCR. We will calculate rebreathed air volume based on people density and CO_2 levels and develop a mathematical model to estimate the risk of TB transmission. The mathematical model can then be used to estimate the effect of possible interventions such as separating patient flows or improving ventilation in reducing transmission. The feasibility of our approach was recently demonstrated in a pilot study in a primary care clinic in Cape Town, South Africa.

Ethics and dissemination: The study was approved by the University of Cape Town (HREC/REF no. 228/2019), the City of Cape Town (ID-8139) and the Ethics Committee of the Canton Bern (2019-02131), Switzerland. The results will be disseminated in international peer-reviewed journals.

For peer review only - http://bmjopen.bmj.com/site/about/guidelines.xhtml

Strengths and limitations of this study

- We describe the protocol for a prospective study design to studying TB transmission in primary care clinics in high TB and HIV burden settings.
- This rapid approach will combine a wide range of different measurements, including patient waiting times and movements, acoustic recording of coughing, measurement of carbon dioxide levels as a natural tracer gas, air humidity and semi-quantitative detection of Mycobacterium tuberculosis particles in the air.
- We will develop a mathematical model which will integrate the collected data to estimate the risk of TB transmission, identify key drivers of transmission and evaluate the impact of infection control measures such as improved ventilation or wearing masks.
- A main limitation of this study design is the lack of direct observation of transmission events and the reliance on the number of Mycobacterium tuberculosis particles in the air as a proxy for TB transmission.
- Study limitations pertain to the need for stable electricity and WIFI for data collection.

For peer review only

For peer review only - http://bmjopen.bmj.com/site/about/guidelines.xhtml

INTRODUCTION

Tuberculosis (TB), caused by the bacterium *Mycobacterium tuberculosis (Mtb)*, remains a major global public health problem, particularly in the context of HIV and drug resistance. Sub-Saharan Africa is one of the most heavily burdened regions globally, although control measures have been in place since the beginning of the 20th century.[1] Over a century of investment in TB control has reduced TB mortality, but effective strategies are urgently needed to reduce TB transmission.[2] Drivers of the TB epidemic in sub-Saharan Africa are HIV-infection and the resulting immunodeficiency (the strongest risk factor),[1] delayed diagnosis and treatment as well as undetected and untreated cases of TB or drug-resistant TB. These factors allow infectious TB patients to transmit *Mtb* to the community.[3-5] There are still many gaps in our knowledge on TB transmission such as the factors and locations associated with the risk of transmission, the effectiveness of infection control measures at clinics in high-burden settings, and the most appropriate intervention strategies to interrupt transmission.[5-7]

For TB transmission to occur, infected individuals must expel *Mtb* bacilli from their respiratory tract, and an uninfected individual must inhale aerosols containing live bacilli to become infected. Transmission of *Mtb* is difficult to measure due to the lack of an *in vitro* test assay. The preferred approach is to measure presumptive transmission resulting in secondary cases as determined by molecular/genomic epidemiology. TB transmission has traditionally been investigated using contact tracing, analyses of geo-temporal clustering, and molecular typing.[8 9] However, molecular methods require resource-intensive culturing of strains and measure only transmission resulting in secondary cases. Furthermore, contact tracing is difficult to implement in resource-limited settings and may not be an effective control strategy in endemic areas where casual contacts are increasingly recognized as an important contributor to transmission.[10] New approaches are therefore urgently needed.

Mtb is carried in airborne particles (called infectious droplets), which are generated when people with TB cough, sneeze or shout.[5] Indoor carbon dioxide (CO_2) levels can be used to assess the amount of exhaled air in a room, and the amount of rebreathed air.[11-13] Humidity is associated with Mtb survival in the air.[14] Viable *Mtb* particles have been captured from contaminated air.[11 15-17] We describe a

For peer review only - http://bmjopen.bmj.com/site/about/guidelines.xhtml

unique study design to assess risk factors for airborne TB transmission in primary care clinics in high TB/HIV burden settings. The approach combines a range of relevant measurements, including data on patient and infrastructure, movements of patients through the facility, coughing, environmental indoor CO_2 levels, and concentration of *Mtb* particles in the air.

We hypothesize that (i) exposure to *Mtb* particles at the clinic can be estimated by studying the patient flow and CO_2 levels; (ii) the number of individuals present, the rebreathed fraction and the frequency of coughs influences exposure to *Mtb*; (iii) clinics with small and crowded waiting rooms, low ventilation, and suboptimal patient separation have an increased risk of TB transmission.

METHODS

Study design

Longitudinal study with data collection at the levels of the patients, the clinic, and the environment. Data collection will take place over four weeks. Figure 1 shows the floor plan of an exemplary primary clinic with the planned study activities, including the recording of patient movements coughing, CO_2 levels and *Mtb* particles in the air.

Study setting and study population

The study will take place at several ART or primary care clinics in countries participating in the International Epidemiology Database to Evaluate AIDS in Southern Africa (IeDEA-SA) collaboration with a high TB and HIV burden. These clinics are located in urban and peri-urban communities with predominantly young and black African residents. TB and HIV are both prevalent in these communities.

Patient-level variables

Video sensor data

A person-tracking sensor system developed by Xovis (Zollikofen, Switzerland and Cambridge, MA; see www.xovis.com) will be used to monitor the clinic attendees' movements. The data will be used to calculate waiting times, the number of people in different locations, and the average distance between people and to identify highly frequented areas. Several sensors will be installed to cover the clinic area, calibrated

For peer review only - http://bmjopen.bmj.com/site/about/guidelines.xhtml

For peer review only

and validated. Sensors with overlapping ranges will be combined for the seamless coverage of people's movements over large areas (Figure 2). The raw data consist of the person's height, time, date, and the position (x-y coordinates) for each unique individual during the duration of their stay within the clinic. The data are captured every 0.25 seconds. The raw data are then parsed by a python script to calculate the height, total movement, and observation time at different locations, and to visualize hotspots.

The sensors have four levels of privacy. In our study, privacy will be set to level 2, which means that the data are a fully anonymized stream of the coordinates of moving dots. These dots will be linked probabilistically to the anonymized clinical data thus excluding any risk of re-identification. Further, the images of tracked individuals taken by the sensors will not be stored (see also the data privacy and security statement by Xovis).[18 19]

Patient and clinic data

We will extract clinical data from the electronic patient registry for all patients who visited the clinics during the four weeks of data collection. The data will include date and time of registration, age, sex, height (used for linkage with video sensor data), HIV status, presumptive TB ("TB suspects"), mode of TB diagnosis (sputum smear microscopy, Xpert MTB/RIF, mycobacterium culture), TB diagnostic test results, date of TB treatment start, current anti-TB treatment regimen. We will not extract any personal data, such as names or social security numbers. The clinic-level data will include information such as the setting (urban or rural), level of care, the number of patients seen each year, availability of adult and paediatric care, TB control measures (natural ventilation, use of masks, separation of TB patients or coughers), and the floor plan of the clinics. The data will be collected using the web-based REDCap data entry system (www.redcap.org).[20]

Definitions

Presumptive TB refers to a patient who presents with clinical symptoms or signs suggestive of TB. A bacteriologically confirmed TB case is a person for whom a biological specimen is positive by smear, microscopy, culture or rapid diagnostic such as Xpert MTB/RIF or line probe assay (LPA).[21]

Environmental variables

For peer review only - http://bmjopen.bmj.com/site/about/guidelines.xhtml

CO_2, relative humidity and temperature monitoring

We will use CO_2 monitors (Digital CO2 Monitor Carbon Dioxide Meter XE-2000, XEAST, Guangdong, China) which include the COZIR-A sensor for ambient CO_2 levels of 0–1% (Gas Sensing Solutions, Cumbernauld, Scotland). The monitors will record indoor CO_2 concentrations (in parts per million [ppm]), temperature, and relative humidity (RH) at minute intervals. Data from the five monitors are stored and exported in serial digital format.[15 16] At each clinic we will install five monitors to cover the most visited spaces and a control area outside the clinic (Figure 1). The monitors auto-calibrate over time to a standard minimum value of 400 ppm. This value is close to the average monthly outdoor carbon dioxide concentration measured at Cape Point, South Africa (405.48 ppm in December 2018).[22] Monitors will be run for one week before the start of the study to allow them to calibrate and to confirm that they delivered comparable data.

Cough monitoring

We will install a microphone (RØDE NT-USB, Sydney, Australia) near the ceiling to continuously record the sounds in the waiting room (Figure 1). *CoughSense*, a deep learning cough detection algorithm based on MXNet, an open-source deep-learning software framework, was developed to classify audio signals as coughing or other sounds.[23] The algorithm uses spectrograms extracted from the raw audio for classification. The model was trained and tested using multiple audio recordings obtained through clinical and ambulatory deployments. We will identify audio records with cough sounds and calculate the cough frequency, intensity and duration. The cough data will be linked with the CO_2 data and video senor data by time and date. Finally, we will probabilistically link the video sensor data (i.e. the moving dots) with the clinical data by the height of the patient, time and date of the visit.

Bio-aerosol sampling and molecular detection

We will collect *Mtb* particles from the air using mobile bio-aerosol sampling devices (Dry Filter Unit [DFU]-1000, Lockheed Martin Integrated Systems, Gaithersburg, MD, USA). The DFU 1000 is a portable biological air sampler. Ambient air is drawn through 1 µm polyester-felt filters at a rate of ~1000 l/min via an electrical blower. The DFU is a useful collection system for low concentration aerosols, capturing particles of 1 µm or larger, and allowing easy access for the retrieval of filters. One DFU will

For peer review only - http://bmjopen.bmj.com/site/about/guidelines.xhtml

be placed in the waiting room and the other in the TB treatment room (Figure 1). Each DFU collects air for 7 hours (two periods of 3.5 hours) every day onto two filters. Due to logistical and human resource limitations, we will be able to change filters only twice per day. Therefore, we decided to run the mobile bio-aerosol sampling devices during the busiest time at the clinic, between 7 am and 2 pm. All other data will be collected from 7 am to 4 pm.

As previously described,[17] duplicate filters from each sampling session will be transferred to 50mL Falcon tubes and vortexed in sterile phosphate buffered saline (PBS) (with 0.05% Tween 80). Following centrifugation at 3750 rpm for 15 mins, filters will be removed and the pellet subjected to DNA extraction. DNA from *Mtb* cells will be extracted using an in-house lysis buffer with subsequent pelleting (centrifugation 13000 rpm for 10 mins) of DNA and resuspension in 50 µl of Tris-EDTA buffer (10mM Trist, 1mM EDTA, pH 8.0). Given that ddPCR is relatively robust against inhibitors, no further DNA purification will be required. The primer/probe combinations and reaction conditions for *Mtb* specific ddPCR have been described.[16] Samples with known amounts of purified *Mtb* DNA (0.01ng and 0.001ng) will be included to serve as positive and nuclease-free water as negative control. The data generated from the ddPCR reaction will be analyzed via the Umbrella pipeline, [24] using wells with a minimum of 10,000 droplets.

Statistical analyses

We will describe the data and examine associations between sources of data and then use results to parameterize a mathematical model (Table 1). The statistical analysis will quantify the joint association between the clinical and environmental variables and the number of *Mtb* particles measured by the mobile aerosol sampling. To this end, we will use Poisson regression, with the number of *Mtb* particles by the period of time as the dependent variable, considered as a proxy measure for the risk of TB transmission. As this variable is measured by periods of 3.5 hours, the other variables will be aggregated over the same periods. We will consider all combinations of independent variables, including second-order interactions, and compare the model using standard model selection methods. We will use variable selection methods in a Bayesian framework, including the deviance information criterion (DIC) and the leave-one-out information criterion (LOOIC).[25]

We will operationalize the variables as follows:

For peer review only - http://bmjopen.bmj.com/site/about/guidelines.xhtml

1. *Patient data*: Numbers and characteristics of patients consulting the clinic overall will be summarized using descriptive statistics.
2. *Video sensor data:* Raw data about indiviudals movements will be transformed into waiting times until medical consultation, number of individuals in the different locations, highly frequented areas in the clinic and the average distance between patients (i.e. clustering). We will link each tracked individual to the clinical data collected from the clinic's database (i.e. TB/HIV diagnosis), using the order of arrival and time of registration.
3. *CO_2 data*: Measurements of CO_2 concentration at the different locations in the clinic (waiting room, registration desk, TB treatment room), together with estimates in outdoor air, will be used to estimate the proportion of air in the different locations that was expired by individuals, the *rebreathed fraction*.[12] We will use a modified Wells-Riley model appropriate for non-steady states conditions of ventilation and number of individuals to describe and calculate the shared rebreathed air in the different locations in either liter/minute or liter/day and the air exchange (liter/hour per person). Table 2 describes the parameters to calculate the shared rebreathed air and the air exchange.[13 16 26 27]

The proportion of rebreathed air (*f*) will be calculated from the excess CO_2 measured indoors, divided by the exhaled CO_2 *(Ca)*:

Equation 1:

$$f = \frac{(C - Co)}{Ca}$$

(see Table 1 for definitions of parameters in this and the following equations).

Before we can calculate the rebreathed air volume (RAV) we will need the rebreathed proportion from other people (fo). Therefore we need to know the number of people *(n)* present at each time point at the clinic:

Equation 2:

$$fo = f \times \frac{(n-1)}{n}$$

For peer review only - http://bmjopen.bmj.com/site/about/guidelines.xhtml

Finally, we can calculate the RAV for each minute by multiplying fo and the minute respiratory volume (8 liters per minute, *[p]*) as:

Equation 3

$$Rebreathed\ air\ volume\ (RAV) = (pfo)$$

In the next step, we aim to calculate the ventilation rate (air exchange). The indoor CO_2 generated rate is the product of the average volume of gas exhaled per person (0.13 l/s per person*, [V]*) and the CO_2 concentration of the exhaled air:

Equation 4:

$$Indoor\ CO_2\ generation\ rate\ in\ l/s\ per\ person\ (G) = VxCa$$

The ventilation rate is expressed as:

Equation 5:

$$Ventilation\ rate\ in\ l/s\ per\ person\ (Q) = \frac{G}{C - Co}$$

To calculate the air exchange per hour (ACH, *Equation 6*) we need to know the volume of air in a given location.

4. *Relative humidity and temperature:* We will describe changes in relative humidity and temperature over the day.
5. *Cough sounds*: Frequency, intensity, duration of recorded coughs per time period.[28 29]
6. *Detection of Mtb in bio-aerosol sampling*: The number of *Mtb* genome copies present in each sample.

Mathematical modelling

A mathematical model (Figure 3) will integrate all sources of data to model the risk of TB transmission and identify key drivers of transmission.[13 26 30 31] We hypothesize that the number of individuals present, the rebreathed fraction and the frequency of coughs will have the greatest influence on the risk. As we do not observe transmission events, we will use *yu*, the number of *Mtb* genomes counted by the bio-aerosol sampling for each 6-hour period *u*, as the dependent variable. The model will describe *yu* as a Poisson process with time-dependent intensity *λt* (also called a Cox process). This

For peer review only - http://bmjopen.bmj.com/site/about/guidelines.xhtml

intensity can be interpreted as a proxy for the (unobserved) risk of TB transmission. We will model *λtu* using 10-minutes time periods *t* within *u*. In a first simple model, *λtu* will integrate multiple independent variables *xtu*: the number of individuals in the waiting room during time *t*, the rebreathed fraction and the number of coughs. The model will be estimated in a Bayesian framework using *Stan*, a probabilistic programming language.[32]

We will use several metrics to assess the goodness of fit of the model. We will then add more complexity to the model by integrating other independent variables such as the characteristics of the patients, shared rebreathed air (CO_2 monitors),[12 13 15 16] patient flow (clustering of individuals, movements of patients), and other potential drivers (intensity/frequency/duration of coughs).[28 29]

Having developed the model of the risk of TB transmission, we will evaluate the possible effect of interventions such as limiting the number of patients in the waiting room, separating coughers or increasing ventilation. All analyses will be performed in R (version 3.6.0) or STATA (version 15.1, Stata Corporation, Texas, USA).

Reporting

The results from this study will be reported following the recommendations of the STROBE statement.[33]

Pilot study

We conducted a pilot study in Cape Town (South Africa) to examine the feasibility of our approach (Figure 1). The pilot study took place at a primary care clinic, which incorporates HIV counselling and testing, and a TB clinic for the diagnosis and management of TB. The clinic is situated in Masiphumelele, a large settlement of formal and semi-formal housing in Cape Town, which has previously been described.[34 35] The clinic is open on workdays from Monday till Friday from 7 am till 4 pm (opening hours of the clinic). We studied clinic activities over four weeks on workdays between July 25 and August 23 2019.

Data collection was successful overall, however, we experienced several power cuts during the pilot study. An important lesson learned is that power banks for laptops and WiFi routers are needed, as well as UPS power stabilizers and access to a generator as back-up for the DFU and CO_2 monitors.

For peer review only - http://bmjopen.bmj.com/site/about/guidelines.xhtml

Patient and public involvement

We discussed the aims and design study with local clinic staff, colleagues at the University of Cape Town and public health specialists early on in the planning phase and developed the specific objectives and data collection procedures in collaboration with them. Patients were not involved in the design, recruitment or conduct of the study. We will make the results of this study available to the participating clinics and the public health authorities.

DISCUSSION

TB control is particularly relevant for sub-Saharan Africa, which carries a disproportionally large portion of the global burden of both TB and HIV. There is an urgent need to understand the drivers of TB transmission to reduce TB incidence using new intervention approaches.[1] A better understanding of transmission, coupled with a rapid test system are likely to contribute to improving TB control in clinic settings. This project will provide new insights into the complex TB transmission framework at a primary care clinic in an endemic setting using clinical data, CO_2 levels, cough analyses, video tracking and *Mtb* particles sampled from the air. Although the large-scale implementation and evaluation of interventions are beyond the scope of this study, the results will generate new hypotheses and opportunities for public health intervention studies (e.g., randomized controlled or cluster-randomized trials).

Importantly, with mathematical models based on real-life data, we can evaluate the likely effect of interventions thus improving intervention studies, and inform help with logistical and infrastructural planning of primary care clinics to reduce the transmission risk. Having established feasibility in one clinic in South Africa, we are planning to collect data in several countries in sub-Saharan Africa. The results from this broader study have the potential to inform national and international guidelines to reduce TB transmission at health care centres.

Strengths and limitations: This is a novel and rapid approach to studying TB transmission combining a wide range of different measurements, which goes beyond

For peer review only - http://bmjopen.bmj.com/site/about/guidelines.xhtml

the traditional methods such as contact tracing, geo-temporal clustering or molecular genotyping. It will lead to a comprehensive transmission model to measure the effects of various interventions in the clinic setting, paving the way for future studies. Study limitations pertain to the need for stable electrical power and WiFi over 24 hours for data collection at the primary care clinic, which is often an issue in low- and middle-income countries. We will address this problem by using UPS power stabilizers with access to generator back-up power.

ETHICS STATEMENT AND DISSEMINATION

The University of Cape Town Faculty of Health Sciences Human Research Ethics Committee (HREC/REF: 228/2019), the City of Cape Town (Project ID 8139) and the Ethics Committee of the Canton of Bern (2019-02131), Switzerland approved the pilot study. Expansion of the study will include other clinics in the greater Cape Town metropolitan area, but also clinics in Zambia, Zimbabwe or Malawi, including urban and rural sites to ensure representative and generalizable results. Separate ethical approval will be sought from the relevant ethics committees or institutional review boards.

The results will be disseminated in international peer-reviewed journals and presented at national and international conferences.

AUTHORS CONTRIBUTIONS

Overall concept: KZ, CM, MB, RW, ME, LF. Medical informatics: SB, JR. Cough sound concept: XL, MS, CM, RW. Video sensor concept: KZ, JR, SB, ME, LF. Patient data concept: CM, KM, RW. Laboratory work concept: CM, AK, KM, DW, RW. Modelling work concept: KZ, JR, SB, ME, LF. Pilot study coordination: CM, KM, RW. KZ, MB, ME, LF wrote the first draft of the paper, which was reviewed by all authors and revised on the basis of the comments received by co-authors. All authors approved the final version of the manuscript.

ACKNOWLEDGEMENTS

For peer review only - http://bmjopen.bmj.com/site/about/guidelines.xhtml

We would like to thank Mbali Mohlamonyane for setting up the bio-aerosol sample collection system, Zeenat Hoosen and Ronnett Seldon for developing the laboratory protocols. We are also grateful to the clinical manager,the staff members at the clinic and all patients who contributed to this study. Finally, we would like to thank for the support from the City of Cape Town for the use of one of their clinic facilities. Research findings and recommendations do not represent an official view of the City of Cape Town.

SOURCES OF SUPPORT

The roll-out of this study is supported by a grant from the Swiss National Science Foundation (grant no. CRSK-3_190781). KZ and LF are supported by the National Institute of Allergy and Infectious Diseases (NIAID) through grant no. 5U01-AI069924-05, ME by special project funding (grant no. 174281) from the Swiss National Science Foundation.

COMPETING INTERESTS

All authors declare that they have no conflicts of interest.

For peer review only - http://bmjopen.bmj.com/site/about/guidelines.xhtml

REFERENCES

1. World Health Organization. Global tuberculosis report 2018. *World Health Organization Document* 2018;WHO/HTM/TB/2018:277.
2. Hermans S, Horsburgh CR, Jr., Wood R. A Century of Tuberculosis Epidemiology in the Northern and Southern Hemisphere: The Differential Impact of Control Interventions. *PloS one* 2015;10(8):e0135179. doi: 10.1371/journal.pone.
3. Wood R, Lawn SD, Caldwell J, et al. Burden of new and recurrent tuberculosis in a major South African city stratified by age and HIV-status. *PloS one* 2011;6(10):e25098. doi: 10.1371/journal.pone.0025098
4. Anonymous. Tuberculosis: a much-needed focus on transmission. *Lancet Respir Med* 2019;7(6):465. doi: 10.1016/S2213-2600(19)30148-1
5. Rieder HL. Epidemiologic basis of tuberculosis control. In: International Union Against Tuberculosis and Lung Disease, ed. 1 ed. Paris: International Union Against Tuberculosis and Lung Disease, 1999:1-162.
6. Lin HH, Dowdy D, Dye C, et al. The impact of new tuberculosis diagnostics on transmission: why context matters. *Bull World Health Organ* 2012;90:739-47A.
7. Middelkoop K, Mathema B, Myer L, et al. Transmission of tuberculosis in a South African community with a high prevalence of HIV infection. *J Infect Dis* 2015;211:53-61.
8. Walker TM, Ip CL, Harrell RH, et al. Whole-genome sequencing to delineate *Mycobacterium tuberculosis* outbreaks: a retrospective observational study. *The Lancet Infect. Dis* 2012 doi: S1473-3099(12)70277-3 [pii];10.1016/S1473-3099(12)70277-3 [doi]
9. Cook VJ, Shah L, Gardy J, et al. Recommendations on modern contact investigation methods for enhancing tuberculosis control. *IJTLD* 2012;16:297-305.
10. Middelkoop K, Bekker LG, Morrow C, et al. Decreasing household contribution to TB transmission with age: a retrospective geographic analysis of young people in a South African township. *BMC Infect Dis* 2014;14:221. doi: 10.1186/1471-2334-14-221
11. Andrews JR, Morrow C, Walensky RP, et al. Integrating social contact and environmental data in evaluating tuberculosis transmission in a South African township. *J Infect Dis* 2014 doi: 10.1093/infdis/jiu138

For peer review only - http://bmjopen.bmj.com/site/about/guidelines.xhtml

12. Myatt TA, Johnston SL, Rudnick S, et al. Airborne rhinovirus detection and effect of ultraviolet irradiation on detection by a semi-nested RT-PCR assay. *BMC public health* 2003;3:5.

13. Rudnick SN, Milton DK. Risk of indoor airborne infection transmission estimated from carbon dioxide concentration. *Indoor Air* 2003;13(3):237-45.

14. Sornboot J, Aekplakorn W, Ramasoota P, et al. Detection of airborne Mycobacterium tuberculosis complex in high-risk areas of health care facilities in Thailand. *IJTLD* 2019;23(4):465-73. doi: 10.5588/ijtld.18.0218

15. Wood R, Morrow C, Ginsberg S, et al. Quantification of shared air: a social and environmental determinant of airborne disease transmission. *PloS one* 2014;9(9):e106622. doi: 10.1371/journal.pone.0106622

16. Richardson ET, Morrow CD, Kalil DB, et al. Shared air: a renewed focus on ventilation for the prevention of tuberculosis transmission. *PloS one* 2014;9(5):e96334. doi: 10.1371/journal.pone.0096334

17. Patterson B, Morrow C, Singh V, et al. Detection of Mycobacterium tuberculosis bacilli in bio-aerosols from untreated TB patients. *Gates Open Res* 2017;1:11. doi: 10.12688/gatesopenres.12758.1

18. Xovis. Guaranteed data privacy Zollikofen2018 [Available from: https://www.xovis.com/technology/detail/guaranteed-data-privacy/ accessed 03.12.2019 2019.

19. Xovis. Xovis' Privacy Policy Zollikofen2018 [Available from: https://www.xovis.com/data-privacy/ accessed 03.12.2019 2019.

20. Harris PA, Taylor R, Minor BL, et al. The REDCap consortium: Building an international community of software platform partners. *J. Biomed. Inform* 2019;95:103208. doi: 10.1016/j.jbi.2019.103208

21. World Health Organization. Definitions and reporting framework for tuberculosis– 2013 revision: World Health Organization 2013.

22. National Oceanic & Atmospheric Administration. Earth System Research Laboratory; Global Monitoring Division [updated 09.11.2019. Available from: https://www.esrl.noaa.gov/gmd/dv/data/index.php?site=CPT&parameter_name=Carbon%2BDioxide,%20accessed%2012%20November%202019 accessed 12.11.2019 2019.

23. Chen T, Li M, Li Y, et al. Mxnet: A flexible and efficient machine learning library for heterogeneous distributed systems. *arXiv preprint arXiv:151201274* 2015

For peer review only - http://bmjopen.bmj.com/site/about/guidelines.xhtml

24. Jacobs BKM, Goetghebeur E, Vandesompele J, et al. Model-Based Classification for Digital PCR: Your Umbrella for Rain. *Anal. Chem* 2017;89(8):4461-67. doi: 10.1021/acs.analchem.6b04208
25. Vehtari A, Gelman A, Gabry J. Practical Bayesian model evaluation using leave-one-out cross-validation and WAIC. *Stat. Comput* 2017;27(5):1413-32.
26. Riley RL, Mills CC, Nyka W, et al. Aerial dissemination of pulmonary tuberculosis. A two-year study of contagion in a tuberculosis ward. 1959. *Am J Epidemiol* 1995;142(1):3-14. doi: 10.1093/oxfordjournals.aje.a117542
27. Wells WF. On air-borne infection*: Study II.droplets and droplet nuclei. *Am J Epidemiol* 1934;20(3):611-18. doi: 10.1093/oxfordjournals.aje.a118097
28. Birring SS, Fleming T, Matos S, et al. The Leicester Cough Monitor: preliminary validation of an automated cough detection system in chronic cough. *Eur Respir J* 2008;31(5):1013-8. doi: 10.1183/09031936.00057407
29. Botha GHR, Theron G, Warren RM, et al. Detection of tuberculosis by automatic cough sound analysis. *Physiol Meas* 2018;39(4):045005. doi: 10.1088/1361-6579/aab6d0
30. Gammaitoni L, Nucci MC. Using a mathematical model to evaluate the efficacy of TB control measures. *Emerg Infect Dis* 1997;3(3):335-42. doi: 10.3201/eid0303.970310
31. Issarow CM, Mulder N, Wood R. Modelling the risk of airborne infectious disease using exhaled air. *J. Theor. Biol* 2015;372(0):100-06. doi: http://dx.doi.org/10.1016/j.jtbi.2015.02.010
32. Carpenter J, Crutchley P, Zilca RD, et al. Correction: Seeing the "Big" Picture: Big Data Methods for Exploring Relationships Between Usage, Language, and Outcome in Internet Intervention Data. *J Med Internet Res* 2017;19(12):e347. doi: 10.2196/jmir.8099
33. Vandenbroucke JP, von Elm E, Altman DG, et al. Strengthening the Reporting of Observational Studies in Epidemiology (STROBE): Explanation and Elaboration. *PLOS Med* 2007;4(10):e297. doi: 10.1371/journal.pmed.0040297
34. Wood R, Middelkoop K, Myer L, et al. Undiagnosed tuberculosis in a community with high HIV prevalence: implications for tuberculosis control. *Am J Respir Crit Care Med* 2007;175(1):87-93.

For peer review only - http://bmjopen.bmj.com/site/about/guidelines.xhtml

35. Middelkoop K, Bekker LG, Myer L, et al. Antiretroviral therapy and TB notification rates in a high HIV prevalence South African community. *J. Acquir. Immune Defic. Syndr (1999)* 2011;56(3):263-9. doi: 10.1097/QAI.0b013e31820413b3

36. Fernstrom A, Goldblatt M. Aerobiology and its role in the transmission of infectious diseases. *J Pathog* 2013;2013:493960. doi: 10.1155/2013/493960

37. Emmerich SJ, Persily AK. State-of-the-art review of CO2 demand controlled ventilation technology and application: Citeseer 2001.

For peer review only

TABLES AND FIGURE

Table 1: Description of the measurements

Data source	Parameter	Description	Unit	Measurement taken by:
CO_2 monitor	CO_2	Observed CO_2 concentration in the indoor air per minute and a control in the outdoor air. Based on CO_2 levels (parts per million [ppm]) and people density, we will calculate rebreathed air volume (RAV), which is used as a proxy for airborne TB transmission.[16]	ppm	minute and date
	Relative humidity	Data on the effects of relative humidity on the survival of airborne bacteria are inconsistent.[27 36] However, a recent study found that relative humidity above 65% is associated with *Mtb* survival in the air.[14]	%	minute and date
	Temperature	Temperatures above 24°C are required to reduce airborne bacteria survival.[27 36]	°C	minute and date
Cough recording	Frequency	One of the typical symptoms of TB is coughing; coughing is also the main way of transmission.	n	minute or day and date
	Duration	Duration of each cough is different from healthy and people with TB or other lung diseases [29]	sec	cough by minute and date
	Intensity	Intensity of each cough is different from healthy and people with TB or other lung diseases. [29]	decibel	cough by minute and date
Mobile aerosol sampling	*Mtb* DNA copies	Detection of *Mtb* particles in the air by filter or per day (7am to 2pm).[17]	Copies per microliters	filter (ca.3.5h sampling) or per day
Video sensor	Number of people	From the raw data (x-y coordinates) we can calculate the number of people at a given location by 0.25 seconds and by minute.	n of people	0.25 seconds or minute and by date
	Time spent at a given location	From the raw data (x-y coordinates) we can calculate for each person their time spent at different locations.	minutes	minute and date
Patient charts	Number of registered patients	All patients who are visiting the clinic are registered.	n of registered patient	minute and day
	Number of presumptive TB and of TB patients	From all registered patients we will know the number of presumptive TB and of TB patients.	n of presumptive TB and TB patients	minute and day

For peer review only

For peer review only - http://bmjopen.bmj.com/site/about/guidelines.xhtml

Table 2: Description of the variables to calculate the shared rebreathed air volume and air exchange as well as the parameters to construct the mathematical transmission model.

Parameter	Description	Value
C	Observed CO_2 concentration in the indoor air per minute	observed
Co	CO_2 concentration in the outdoor air per minute	400-420ppm[22]
Ca	CO_2 concentration in the exhaled air	38000-40000ppm[13 37]
f	Proportion of rebreathed air	equation
n	Number of people recorded at the location	observed
fo	Rebreathed proportion from other people	equation
p	Minute respiratory volume	8 l/per minutes[37]
RAV	Rebreathed air volume	equation
V	Average volume of gas exhaled per person	0.13 l/s per person[16]
G	Indoor CO_2 generation rate (l/s per person)	equation
Q	Ventilation rate (l/s per person)	equation
vol	Volume of the room	calculated

For peer review only - http://bmjopen.bmj.com/site/about/guidelines.xhtml

Figure 1: Floor plan of the pilot primary care clinic in Cape Town, South Africa, indicating study measurements.

Figure 2: Output from videao sensors with moving dots showing the tracked persons. The numbers in the dots indicate the height of patients. The different sectors covered by the sensors are merged. Images from the pilot study.

Figure 3: Model structure.

For peer review only

For peer review only - http://bmjopen.bmj.com/site/about/guidelines.xhtml

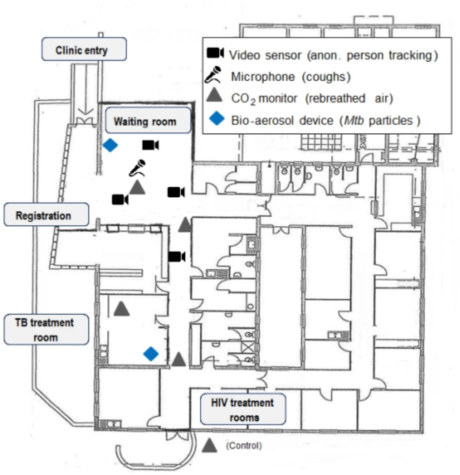

Figure 1: Floor plan of the pilot primary care clinic in Cape Town, South Africa, indicating study measurements

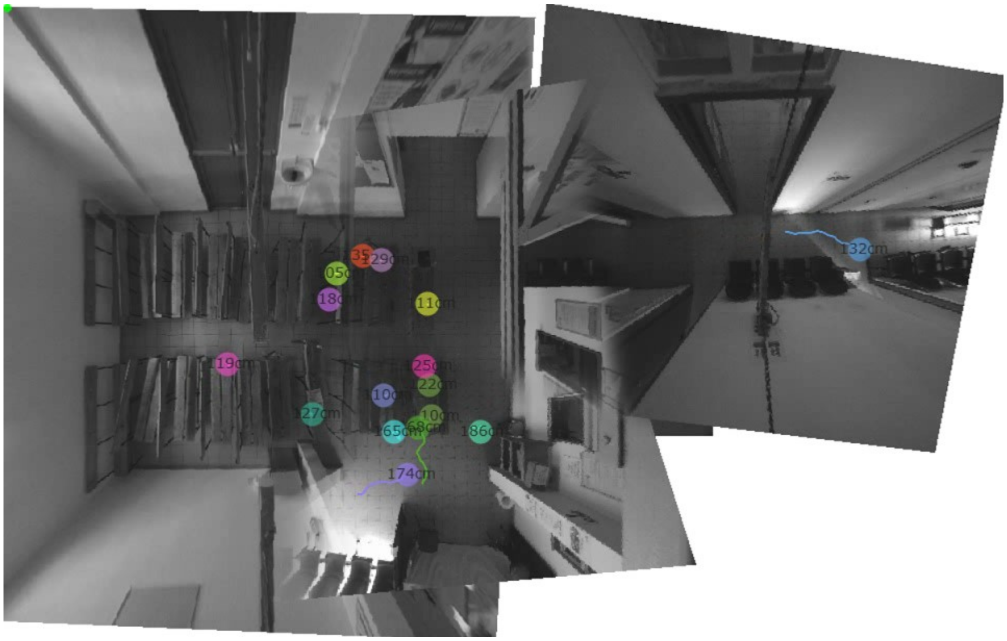

Figure 2: Output from videao sensors with moving dots showing the tracked persons. The numbers in the dots indicate the height of patients. The different sectors covered by the sensors are merged. Images from the pilot study.

81x51mm (300 x 300 DPI)

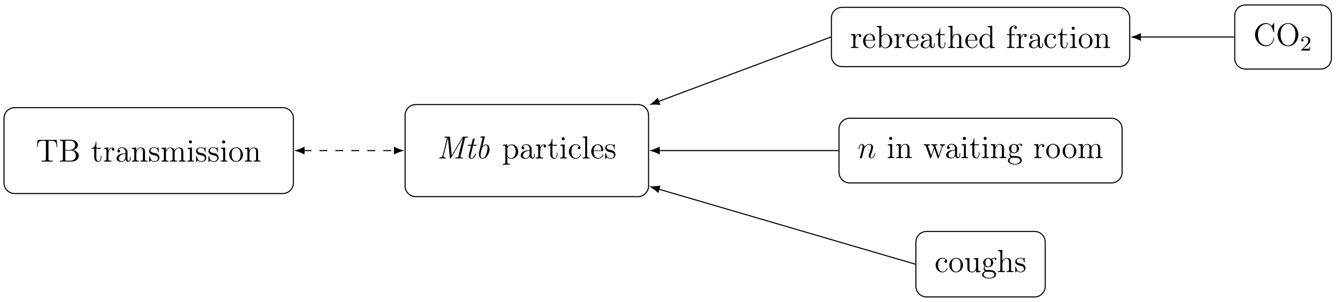

For peer review only
